# ATPase activity tightly regulates RecA nucleofilaments to promote homologous recombination

**DOI:** 10.1038/celldisc.2016.53

**Published:** 2017-01-17

**Authors:** Bailin Zhao, Dapeng Zhang, Chengmin Li, Zheng Yuan, Fangzhi Yu, Shangwei Zhong, Guibin Jiang, Yun-Gui Yang, X Chris Le, Michael Weinfeld, Ping Zhu, Hailin Wang

**Affiliations:** 1 State Key Laboratory of Environmental Chemistry and Ecotoxicology, Research Center for Eco-Environmental Sciences, Chinese Academy of Sciences, Beijing, China; 2 National Laboratory of Biomacromolecules, Institute of Biophysics, Chinese Academy of Sciences, Beijing, China; 3 Key Laboratory of Genomics and Precision Medicine, Beijing Institute of Genomics, Chinese Academy of Sciences, Beijing, China; 4 Department of Laboratory Medicine and Pathology, University of Alberta, Edmonton, AB, Canada; 5 Experimental Oncology, Cross Cancer Institute and University of Alberta, Edmonton, AB, Canada

**Keywords:** RecA, ATPase activity, homologous recombination, strand exchange, capillary electrophoresis, laser-induced fluorescence polarization

## Abstract

Homologous recombination (HR), catalyzed in an evolutionarily conserved manner by active RecA/Rad51 nucleofilaments, maintains genomic integrity and promotes biological evolution and diversity. The structures of RecA/Rad51 nucleofilaments provide information critical for the entire HR process. By exploiting a unique capillary electrophoresis-laser-induced fluorescence polarization assay, we have discovered an active form of RecA nucleofilament, stimulated by ATP hydrolysis, that contains mainly unbound nucleotide sites. This finding was confirmed by a nuclease protection assay and electron microscopy (EM) imaging. We further found that these RecA-unsaturated filaments promote strand exchange *in vitro* and HR *in vivo*. RecA mutants (P67D and P67E), which only form RecA-unsaturated nucleofilaments, were able to mediate HR *in vitro* and *in vivo*, but mutants favoring the formation of the saturated nucleofilaments failed to support HR. We thus present a new model for RecA-mediated HR in which RecA utilizes its intrinsic DNA binding-dependent ATPase activity to remodel the nucleofilaments to a less saturated form and thereby promote HR.

## Introduction

Homologous recombination (HR) is essential for genomic integrity by rescuing collapsed replication forks and repairing DNA double-strand breaks in virtually all organisms [1–4]. Defects in recombination-mediated repair are associated with elevated cancer risk [5, 6]. A central feature of HR is a DNA strand exchange reaction catalyzed by RecA and its homologs, which constitute a family of proteins conserved from bacteria to eukaryotes [7, 8]. To initiate DNA strand exchange, *Escherichia coli* RecA proteins are loaded on replication- or damage-induced single-stranded DNA (ssDNA) gaps [9] to form helical nucleofilaments [10]. The RecA nucleofilaments can further bind to homologous double-strand DNA (dsDNA) molecules, and drive strand exchange [11, 12]. Interestingly, through the use of a non-hydrolyzable ATP analog, ATPγS and a ground or transition state analog, ADP-AlF_4_, it was observed that RecA-saturated nucleofilaments can mediate homology recognition and strand exchange until the product heteroduplex releases upon ATP hydrolysis [13, 14]. These seminal studies engendered a canonical and long-standing hypothesis that ATP-bound, RecA-saturated nucleofilaments mediate strand exchange *in vitro* and *in vivo*. These nucleofilaments adopt a stretched, rigid, under-wound and localized B-DNA-like conformation [15]. Derived from this model, the discontinuities in nucleofilaments would stop strand exchange [16]. Interestingly, RecA-saturated nucleofilaments could also be obtained in the presence of hydrolysable ATP, but generally require the assistance of chemical crosslinking [17] or inhibited ATP hydrolysis [18]. Nonetheless, physiologically relevant ATP hydrolysis can stimulate end disassembly of RecA, implicating a mechanism related to the dynamics of RecA nucleofilaments [19]. These observations led us to speculate whether end disassembly accompanying ATP hydrolysis would generate a partly elastic structure of RecA-unsaturated nucleofilaments. Further, if these RecA-unsaturated nucleofilaments are formed, do they exist only transiently or have a relatively long lifetime? More importantly, why are these nucleofilaments produced through high energy-consuming ATP hydrolytic activity if they are not actively involved in mediating HR? These questions prompted us to re-scrutinize the active RecA nucleofilaments in HR.

In this study, we therefore sought to examine the dynamics, structure and functions of RecA nucleofilaments under physiologically relevant conditions. For this purpose, we developed a novel assay uniquely combining highly efficient capillary electrophoresis (CE) separation with laser-induced fluorescence polarization (LIFP) detection. Because the CE separation is rapid and is carried out under physiological conditions, we could rapidly monitor the physiologically relevant dynamics of RecA-DNA filaments and HR. Moreover, the new assay affords accurate quantitation of complex assembly stoichiometry, which enabled us to identify and characterize dynamic filamentous structures. We were thus able to establish that RecA ATPase hydrolytic activity stimulates the formation of the physiologically relevant RecA nucleofilaments containing mainly unbound nucleotide sites. By combining systematic point mutations of RecA with a bacterial HR reporter assay, we show that the newly identified nucleofilaments are fully compatible with HR in an *in vivo* setting.

## Results

### CE-LIFP assay for detection of RecA-saturated nucleofilaments

We developed a CE-LIFP assay for monitoring RecA nucleofilaments, which was performed on laboratory-built CE-LIFP apparatus (Supplementary Figure S1A–C). A TMR-ss83mer DNA (Supplementary Table S1), which was labeled with tetramethylrhodamine (TMR) at the 5′ end, was used as a substrate for studying RecA nucleofilaments. TMR served as a suitable label for the measurement of both fluorescence intensity and polarization.

Two features of the CE-LIFP assay insure that the assay could be used to obtain information reflecting the original binding stoichiometry of RecA nucleofilaments in the homogenous reaction solution. First, during CE separation (<2.5 min), unbound ATP (the major form of ATP) is initially isolated from the zone of RecA nucleofilaments due to differential charge/mass ratio, and then the bound ATP (minor form) continuously dissociates from RecA nucleofilaments due to fast dissociation kinetics and low affinity for ATP (*K*_d_ ~18 μM) [20]. Therefore, CE separation rapidly removes both unbound and bound ATP away from the RecA-DNA filaments zone. This largely inhibits ATP hydrolysis and avoids a dramatic change in the binding stoichiometry of RecA nucleofilaments caused by ATP hydrolysis-induced end disassembly. To further circumvent artifacts arising from ATP hydrolysis, we optimized the CE-LIFP approach by including Ca^2+^ in the CE separation buffer as a stabilizing additive, which can inhibit residual ATP hydrolysis [18]. Of note, the inhibition only occurs after the sample was injected into the capillary for immediate electrophoresis (fast, ~2.5 min). The preserved nucleofilaments substantially result from the reaction solutions.

**Figure 1:**
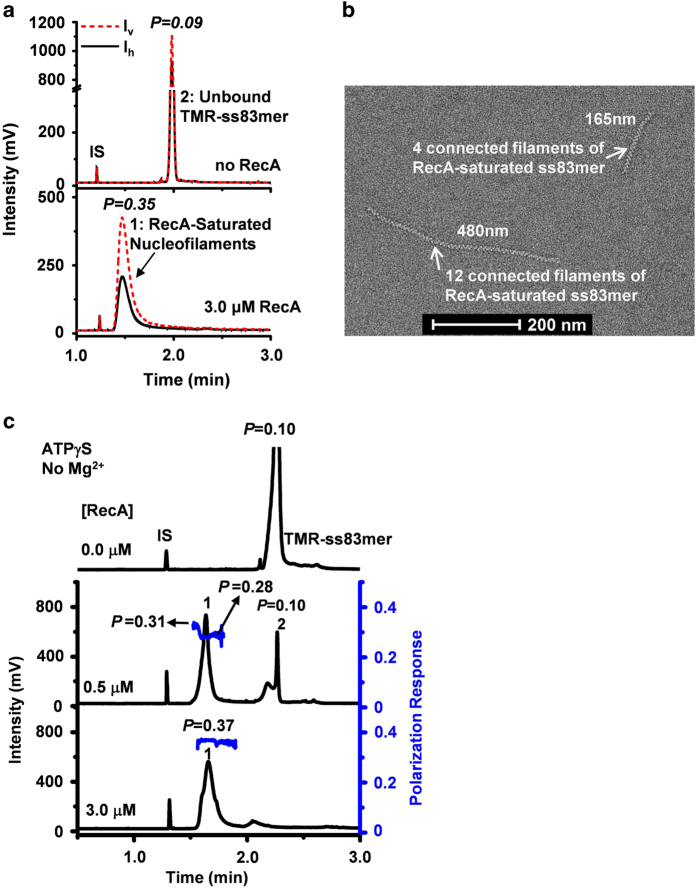
CE-LIFP and EM analysis of the RecA-saturated nucleofilaments stimulated by the non-hydrolyzable ATP analog ATPγS. (**a**) Typical electropherograms of CE-LIFP analysis of RecA assembly on ssDNA (TMR-ss83mer) in the presence of ATPγS (0.1 mM) and Mg^2+^ (10 mM). The reaction solution included 3.0 μM RecA, 10 nM TMR-ss83mer and 0.1 mM ATPγS. *I*_v_ and *I*_h_ represent the intensities of vertically and horizontally polarized fluorescence, respectively. (**b**) EM of the RecA-saturated nucleofilaments stimulated by ATPγS. The reaction solution included 3.0 μM RecA, 300 nM ss83mer and 0.1 mM ATPγS. (**c**) CE-LIFP electropherograms for analysis of the RecA-saturated nucleofilaments in the presence of 0.1 mM ATPγS but absence of Mg^2+^. The reactions also contained 10 nM TMR-ss83mer and 3.0 μM RecA protein, and proceeded at 37 °C for 10 min. IS indicates internal migration marker (**a**, **c**). Peak 1: RecA-saturated nucleofilaments; Peak 2: unbound TMR-ss83mer. Note: (1) In all figures, the concentrations of the oligomers are given in terms of whole oligomer molecules; (2) except in Figure 1a, only *I*_h_ signals are displayed in CE-LIFP electrophoregrams; (3) IS indicates internal migration marker in all figures.

To investigate the reliability of our CE-LIFP approach, we first analyzed RecA nucleofilaments stimulated by a non-hydrolyzable ATP analog ATPγS, conditions under which RecA-saturated nucleofilaments can be obtained [21]. Indeed, as revealed by electron microscopy (EM) imaging, we observed continuous, thin and long filaments (Figure 1b), confirming the formation of the RecA-saturated nucleofilaments. CE-LIFP showed a single peak of RecA nucleofilaments (peak 1, Figure 1a) accompanied by the disappearance of unbound TMR-ss83mer (peak 2, Figure 1a). This complex featured a high polarization value (*P*=0.35). In contrast, unbound TMR-ss83mer (MW 25 kDa) showed only a low polarization response (*P*=0.09). Since the molecular rotation-dependent fluorescence polarization response (ranging from 0 to 0.5, dimensionless) is proportional to the molecular size, the observation of the large polarization value of the RecA-saturated nucleofilaments (*P*=0.35) is consistent with their large molecular weight (MW: 1050 kDa) resulting from the assembly of multiple RecA monomers (~27 monomers; 38 kDa each monomer) on one ss83mer molecule (MW: 25 kDa). Of note, no nucleofilaments other than the RecA-saturated nucleofilaments were observed, and also no unbound TMR-ss83mer was observed (Figure 1a), therefore, the RecA-saturated nucleofilaments are not dissociated during CE separation. This observation suggests that the RecA-saturated nucleofilaments are well preserved throughout CE separation.

We further reduced the binding affinity of ATPγS to the RecA nucleofilaments from *K*_d_: 0.5 μM to *K*_d_: 22 μM by removal of Mg^2+^ in the reactions [20, 22], which is comparable to that of ATP (*K*_d_: 18 μM). Interestingly, we also observed stable RecA-saturated nucleofilaments as the predominant form indicated by a large polarization value (*P*=0.28–0.37) and a large electrophoretic mobility shift from the unbound TMR-ss83mer (Figure 1c). Together, these results validate the CE-LIFP assay as a robust and sensitive means to detect RecA-saturated nucleofilaments.

### Formation of RecA-unsaturated nucleofilaments stimulated by physiologically relevant ATP hydrolysis

**Figure 2:**
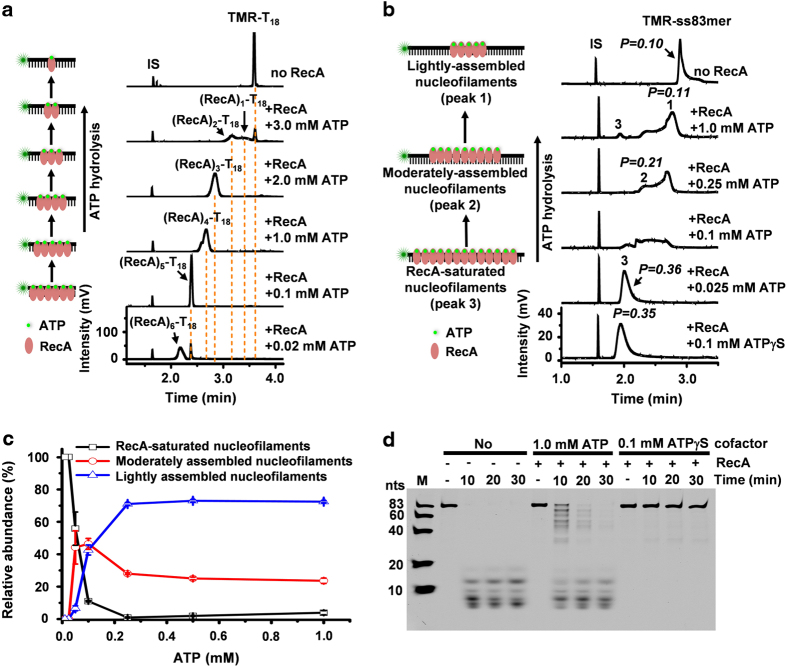
ATP hydrolysis inhibits the formation of the RecA-saturated nucleofilaments and increases the dynamics from nucleation to extension. (**a**) CE-LIF electropherograms of the reaction mixtures of TMR-T_18_ (10 nM) and RecA protein (16.0 μM), showing the formation of six distinct RecA-TMR-T_18_ complexes induced by ATP hydrolysis. The *Y* axis of the bottom trace can be applied to all stacked traces in the panel. (**b**) CE-LIFP electropherograms of the reaction mixtures of TMR-ss83mer (10 nM) and RecA (3.0 μM), showing the formation of three types of RecA nucleofilaments in the presence of ATP. Peaks 1–3 represent the lightly assembled, moderately assembled and RecA-saturated nucleofilaments, respectively. The *Y* axis of the bottom trace can be applied to all stacked traces in the panel. (**c**) The relative abundance of the three types of RecA-TMR-ss83mer filaments is dependent on ATP concentration. (**d**) Denaturing PAGE analysis of time-dependent products of supernuclease digestion of the Cy5-ss83mer (20 nM) and the reaction mixtures of Cy5-ss83mer (20 nM), RecA (3.0 μM) and one nucleotide cofactor as indicated. M indicates ssDNA size markers with a unit of nucleotides (nts). The reactions proceeded at 37 °C for 10 min.

We next sought to examine the assembly of RecA on ssDNA with the involvement of ATP and the full ATPase activity of RecA. First, we examined the initial step of RecA assembly, nucleation, which may cooperatively involve five to six RecA monomers. For this purpose, we used a short oligomer, TMR-T_18_, which would allow up to six RecA monomers to nucleate on the ssDNA, since one RecA monomer binds three nucleotides [15]. It would be anticipated that a single saturated nucleation product, that is, (RecA)_6_-T_18_ complex, should be observed if the cooperative nucleation mechanism seen with non-hydrolyzable ATP analogs [21, 23] is also operative in the presence of ATP. However, in contrast to the above prediction, we observed six well resolved RecA_n_-T_18_ complexes (*n*=1–6), in which one oligo molecule binds one to six RecA monomers (Figure 2a). Similarly, we observed ATP hydrolysis facilitated the formation of five complexes with TMR-T_15_ and four complexes with TMR-T_12_ (Supplementary Figure S2A and B). Significantly, in the presence of a physiologically relevant concentration of ATP (1.0 mM), both TMR-T_15_ and TMR-T_12_ can stably bind two RecA monomers, suggesting nucleation involving two RecA monomers under physiologically relevant conditions (Figure 2a and Supplementary Figure S2). This is consistent with previous work of Knight and his colleague showing the RecA dimer serving as a functioning unit [24]. If the nucleation only requires two RecA monomers, the assembly of RecA on TMR-T_18_ may involve both nucleation and extension since it mainly forms the 1:4 DNA-RecA complex at 1.0 mM ATP (Figure 2a). Of note, these results clearly show that ATP hydrolysis can biochemically stimulate the formation of diverse RecA-ssDNA complexes of distinct stoichiometry.

Understanding the extension of RecA assembly on, we next tested the assembly of RecA on a longer oligomer (TMR-ss83mer), which allowed both nucleation and extension, again using physiologically relevant concentrations of ATP [25]. Interestingly, we observed two new types of RecA-unsaturated nucleofilaments, moderately and lightly assembled nucleofilaments (peaks 2 and 1, Figure 2b), in addition to the RecA-saturated nucleofilaments (peak 3, Figure 2b). In addition, we found that the lightly assembled nucleofilaments predominate with a relative abundance of >70% (Figure 2c) with an increase in ATP concentration to >0.25 mM. Noteworthy, if robust ATP hydrolysis (caused by the injection of 1.0 mM ATP in the reaction solution) during 2–2.7 min CE separation had occurred and resulted in the observation of the lightly assembled nucleofilaments, then robust ATP hydrolysis would have occurred for all the nucleofilaments and we should not have observed any longer nucleofilaments. However, we indeed observed longer filaments (for 1.0 mM ATP) although minor (<5%, Figure 2b and c), excluding the possibility that ATP hydrolysis occurred during CE separation could artificially produce the lightly assembled nucleofilaments.

In the above experiments, Mg^2+^ and ATP were included in the reaction solutions to stimulate the formation of physiologically relevant nucleofilaments, but not included during CE separation. We asked whether Mg^2+^ and ATP are indispensable to preserve the formed nucleofilaments throughout CE separation. So we included Mg^2+^ and ATP for CE separation. Of note, in this experiment, no Ca^2+^ was included in the CE separation buffer. However, we again observed the predominance of the lightly assembled nucleofilaments (Supplementary Figure S3), indicating that the these nucleofilaments are the predominant complexes in the reaction solutions. Furthermore, since we showed that the CE-LIFP approach readily detects RecA-saturated nucleofilaments (Figure 1), the observation of the predominance of lightly assembled nucleofilaments also proves that the RecA-saturated nucleofilaments are not the major class of nucleofilaments.

It is not known whether the exposed ends of short oligonucleotides stimulate rapid RecA dissociation from the nucleofilaments and thus promote the formation of the lightly assembled nucleofilaments. To examine this possibility, we then extended the TMR-ss83mer to an oligonucleotide of 175 nucleotides (TMR-ss175mer). Consistently, we observed the predominance of RecA-unsaturated nucleofilaments (Supplementary Figure S4).

We corroborated these observations using a nuclease protection assay and EM. Supernuclease selectively digested the RecA-bound ss83mer when stimulated by 1.0 mM ATP favoring the lightly assembled nucleofilaments, similar to the unprotected DNA (Figure 2d and Supplementary Figure S5B). However, DNA was not cut when RecA was stimulated by ATPγS or by 0–0.05 mM ATP favoring the RecA-saturated nucleofilaments (Figure 2d and Supplementary Figure S5B). Of note, the nucleotide cofactors themselves do not affect supernuclease digestion activity (Supplementary Figure S5A).

EM analysis clearly showed the saturated nucleofilaments stimulated by ATPγS (Supplementary Figure S6A-(d)) and the presence of the lightly and moderately assembled nucleofilaments, but not the saturated nucleofilaments, when stimulated by 1.0 mM ATP (Supplementary Figure S6A-(b)). Moreover, for single strand DNA plasmid (ΦX174 virion DNA), we also mainly observed short nucleofilaments (30–40 nm, ~67–89 nucleotides) and the absence of the saturated nucleofilaments (Supplementary Figure S6B). The maximum nucleofilament length is only about 90 nm (~200 nucleotides). Essentially, the length of all the observed nucleofilaments (22–200 nucleotides) is far less than the whole length of the tested single strand DNA plasmid (5386 nucleotides) (Supplementary Figure S6C).

Of note, we could observe some RecA bundles induced by the use of 10 mM Mg^2+^ (Supplementary Figure S6A-(a) and -(c)). To avoid the interference of RecA bundling, following previous strategy [26], we used excess ssDNA (300 nM ss83mer or 150 nM ΦX174 circular ssNA). Therefore, the unbound RecA background greatly reduces, and the RecA bundling can be discriminated (Supplementary Figure S6A-(c)) from the isolated RecA filaments (Supplementary Figure S6A-(d)).

**Figure 3:**
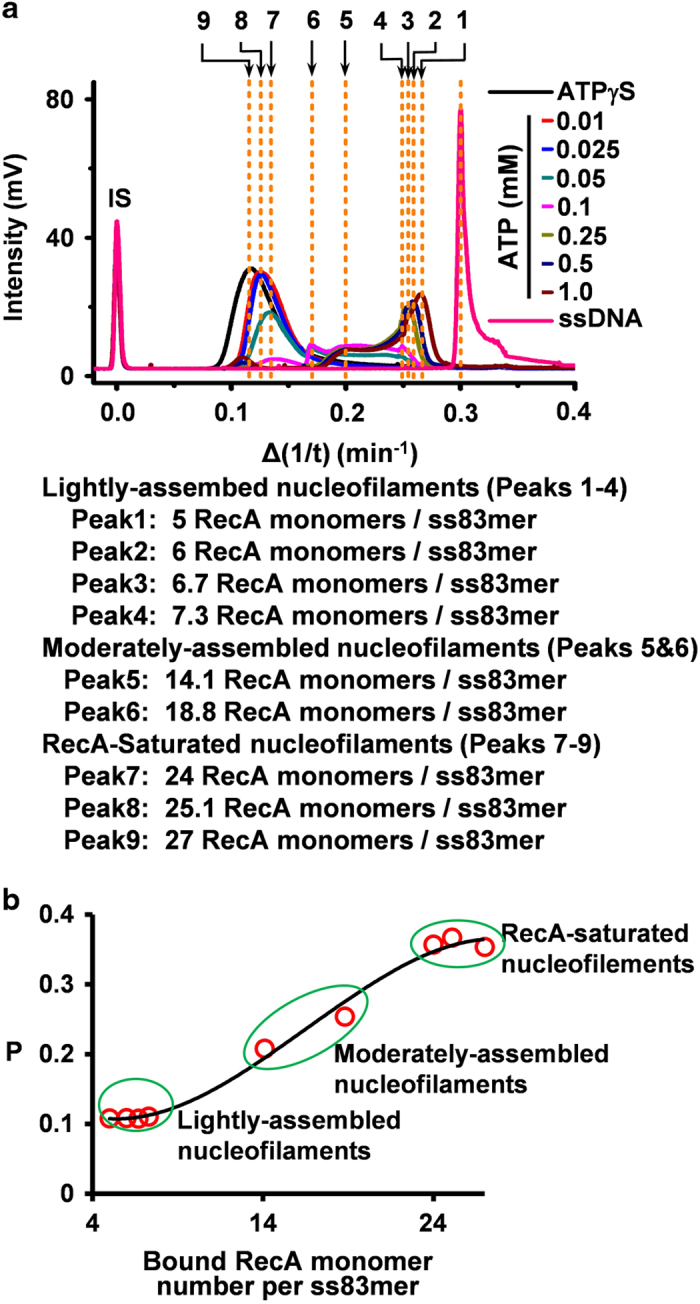
Quantitation of the binding stoichiometry of dynamic RecA nucleofilaments. (**a**) Overlay of the CE-LIFP electropherograms obtained by varying ATP concentration (0.01–1.0 mM) versus Δ(1/t) and the estimated binding number of RecA in each nucleofilament. The RecA-saturated nucleofilaments stimulated by ATPγS and unbound TMR-ss83mer were used to indicate the binding of 27 and 0 RecA monomers to one TMR-ss83mer molecule, respectively. The top numbers (1–9) are ranked in the order of Δ(1/t) for all the peaks of RecA-TMR-ss83mer filaments obtained by the use of ATP at indicated concentrations (0.01–1.0 mM). The binding numbers were estimated using the equation listed in Supplementary Figure S7 and the obtained binding numbers are shown right below the overlapped figures. (**b**) The correlation of the measured polarization values (*P*) of each RecA-TMR-ss83mer filaments with the binding number of RecA monomer per ss83mer (**b**).

We further quantified the binding stoichiometries of various RecA nucleofilaments. The estimation was made by correlation of the reciprocal of the measured migration time of the RecA-ssDNA complexes with their bound-RecA number per oligonucleotide molecule (Supplementary Figure S7). We obtained nine nucleofilaments belonging to the three classes of RecA nucleofilaments by varying the ATP concentrations (0.01–1.0 mM ATP, Figure 3a). We detected 24–27 RecA monomers per ss83mer in the saturated nucleofilaments, and 14–19 RecA monomers per ss83mer in the moderately assembled nucleofilaments, and 5–7 RecA monomers per ss83mer in the lightly assembled nucleofilaments (Figure 3a). The estimated binding number correlated well with the molecular-size-dependent fluorescence polarization response (Figure 3b), confirming the binding stoichiometry measured by CE mobility-shift assay.

Finally, we increased the concentration of RecA monomer from the physiological level (3.0 μM) to 9.0–20 μM. Interestingly, the moderately assembled nucleofilaments (15 RecA monomers/ss83mer for 9.0 μM RecA and 19 RecA monomers/ss83mer for 20.0 μM RecA) were the predominant complexes (Supplementary Figure S8A and B). Even with 20.0 μM RecA, the RecA-saturated nucleofilaments only accounted for 4.0% of the total. The observed moderately assembled nucleofilaments appear to be reasonably stable since a constant *P* value (*P*=0.204–0.206, Supplementary Figure S8C) was observed during a 45 s pause in the CE separation.

Taken together, the above data provide compelling evidence for the predominance of the unbound nucleotides in the active RecA nucleofilaments regulated by physiologically relevant ATP.

### ATP hydrolysis directly promotes the formation of RecA-unsaturated nucleofilaments

In the presence of physiologically relevant ATP, we observed lightly assembled nucleofilaments to be the predominant form of RecA complex, whereas the non-hydrolyzable ATP analog ATPγS only promotes the formation of the RecA-saturated nucleofilaments (Figure 1). Since these results indicated a linkage between ATP hydrolysis and the formation of the active lightly assembled nucleofilaments, we sought more direct experimental proof. As shown in Supplementary Figure S9, RecA can form saturated nucleofilaments without any nucleotide cofactor. However, subsequent addition of ATP (1.0 mM) rendered the RecA-saturated nucleofilaments unstable and they rapidly converted to the lightly assembled nucleofilaments. Moreover, we observed that differential ATP hydrolytic activity of wild type and mutant RecA proteins determine their capability of forming the lightly assembled RecA nucleofilaments (Supplementary Figure S10A and B). Notably, the K72R mutant RecA, which can bind ATP but has almost no ATP hydrolytic activity, failed to mediate the formation of the lightly assembled RecA nucleofilaments (Supplementary Figure S10A). Thus, taken together, our data clearly show a direct requirement for ATP hydrolysis in the formation of the lightly assembled nucleofilaments.

### Lightly assembled nucleofilaments facilitate strand exchange reactions

**Figure 4:**
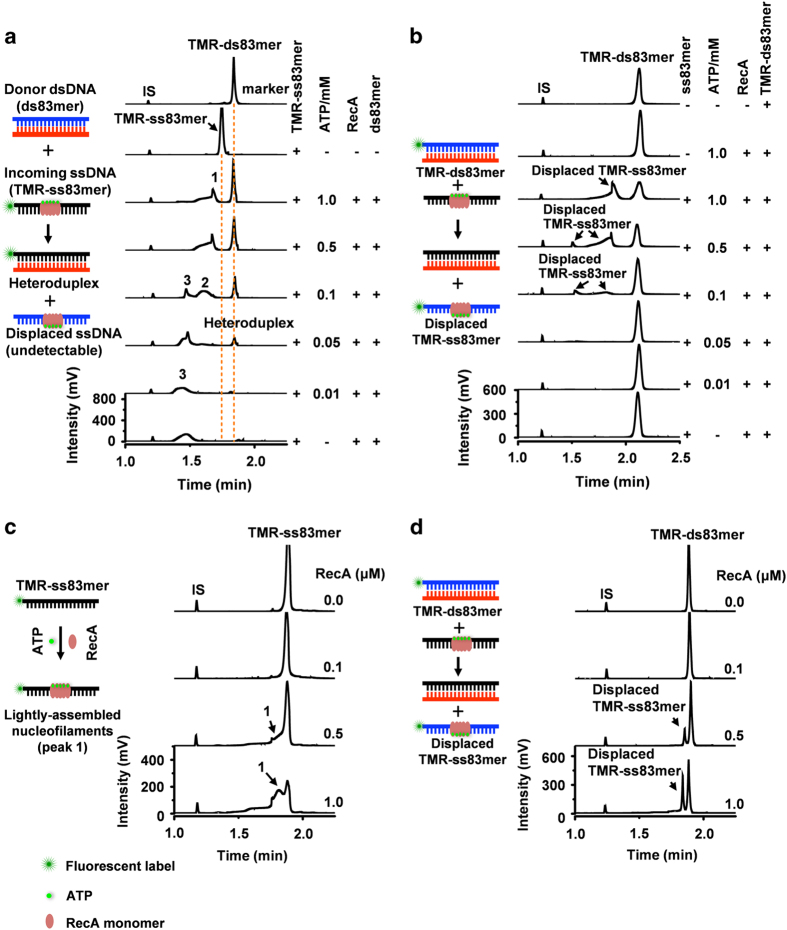
The lightly assembled RecA nucleoflaments facilitate strand exchange. (**a**, **b**) CE-LIFP electropherograms of the products of strand exchange reactions in the presence of ATP. TMR-ss83mer (**a**) or TMR-ds83mer (**b**) was used as the detectable DNA probe to indicate the formation of heteroduplex TMR-ds83mer (**a**) or displaced ssDNA TMR-ss83mer (**b**). The reactions contained 3.0 μM RecA protein, 10 nM ss83mer, 10 nM ds83mer and ATP (0–1.0 mM). (**c**, **d**) CE-LIFP electropherograms of the lightly assembled nucleofilaments (**c**) and the strand exchange reactions (**d**) with RecA at low concentrations (0.1–1.0 μM). TMR-ss83mer (**c**) or TMR-ds83mer (**d**) was used as a fluorescent probe to indicate the formation of detectable lightly assembled nucleofilaments (**c**) or displaced TMR-ss83mer (**d**). ATP was kept at 1.0 mM, and 83mer DNA was kept at 10 nM. Peaks 1–3 represent the lightly assembled, moderately assembled and RecA-saturated nucleofilaments, respectively. The reactions proceeded at 37 °C for 10 min.

Given that lightly assembled nucleofilaments are induced by physiologically relevant ATP, we sought to explore the potential importance of the lightly assembled nucleofilaments in RecA-mediated strand exchange. When a labeled incoming ssDNA (TMR-ss83mer) was incubated with an unlabeled duplex in the presence of RecA (3.0 μM) and stimulated by ATP (0.05–1.0 mM), we observed a newly formed heteroduplex (Figure 4a). The estimated yield of the heteroduplex reached ~42% (0.5–1.0 mM ATP, Supplementary Figure S9A). In contrast, we failed to observe any heteroduplex release when only RecA-saturated nucleofilaments were present (0–0.01 mM ATP) (Figure 4a). When we used the fluorescently labeled donor dsDNA (TMR-ds83mer, see left scheme in Figure 4b) with unlabeled incoming ssDNA, we observed the displaced fluorescent ssDNA, which was originally present in the RecA-bound state, and the concomitant decrease in the TMR-ds83mer (0.05–1.0 mM ATP, Figure 4b). Both the labeling formats showed that the occurrence and completion of strand exchange reactions increased with an increasing ratio of the unsaturated nucleofilaments to the saturated nucleofilaments (Supplementary Figure S11B and C). Gel electrophoresis analysis confirmed this trend (Supplementary Figure S12).

An analysis of the dependence of the strand exchange reaction on RecA concentration revealed that the lightly assembled nucleofilaments were produced down to a RecA concentration of 0.5 μM (Figure 4c), and this was similarly matched by the strand exchange reaction (Figure 4d).

Of note, the presence of Mg^2+^ eliminates any residual RecA-saturated nucleofilaments (Supplementary Figure S13). However, Mg^2+^ is essential for the strand exchange. This observation, therefore, provides further evidence that the RecA-saturated nucleofilaments are not necessary for strand exchange.

### *In vivo* homologous recombination (HR) in *E. coli*

To test RecA activity *in vivo*, we developed an *E. coli* HR assay (Supplementary Figure S14A and for details see Materials and Methods section). In this assay, green fluorescent protein-positive (GFP^+^) cells can be detected only in the designed *E. coli* strains undergoing accurate homology-directed repair. As shown in Supplementary Figure S14B, in response to expression of the rare restriction endonuclease I-SceI, we observed a significantly elevated percentage of GFP^+^ cells for *recA*^*+/+*^ *E. coli*. In contrast, the *recA*^*−/−*^ strain did not generate any GFP^+^ cells, but after complementation with wild-type RecA, we observed a significant recovery of the GFP^+^ cell signal. Again, we did not detect any GFP^+^ cells for the mock plasmid-transformed *E. coli*. These results confirmed the central role RecA plays in the *in vivo E. coli* HR pathway.

**Figure 5:**
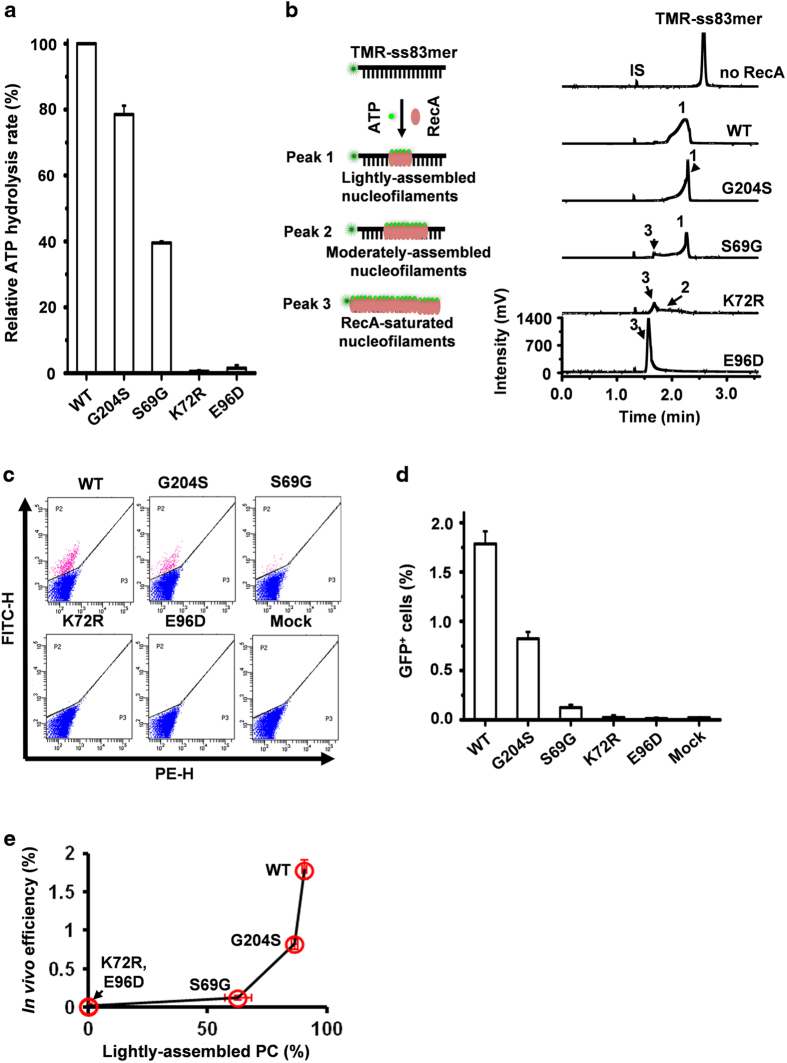
*In vivo E. coli* homologous recombination repair and its correlation with *in vitro* lightly assembled nucleofilaments. (**a**) The ATP hydrolytic activity of four representative RecA mutants (G204S, S69G, E96D and K72R). The activity was normalized to that of the wild type of RecA. The reactions contained 3.0 μM RecA, 10 nM ss83mer and 1.0 mM ATP. (**b**) Electropherograms from CE-LIFP analysis of the mutated RecA-ssDNA filaments. The reactions contained 10 nM TMR-ss83mer, 3.0 μM RecA and 1.0 mM ATP, and proceeded at 37 °C for 10 min. IS indicates internal migration marker. Peaks 1–3 represent the lightly assembled, moderately assembled and saturated nucleofilaments, respectively. (**c**, **d**) FACS analysis of *E. coli* HR efficiency of four RecA mutants (G204S, S69G, E96D and K72R) (**c**) and the corresponding percentage of GFP^+^ cells (**d**). FITC-H and PE-H (**c**) indicate the GFP signal and background fluorescence of the sorted *E. coli* cells, respectively. FITC: ex 488 nm, em 530 nm; and PE: ex 488 nm, em 585 nm. Pink and blue dots (**c**) indicate GFP^+^ and GFP^−^ cells, respectively. (**e**) The correlation of *in vivo* HR efficiency (ref. to **d**) with *in vitro* lightly assembled nucleofilaments as indicated in **b**.

We further examined four representative RecA mutants [27], G204S [28], S69G [29], E96D [30] and K72R [16, 31], which display differential ATP hydrolytic activity (Figure 5a) without sacrificing ATP binding. Notably, RecAG204S, which generates only the lightly assembled nucleofilaments *in vitro* (Figure 5b), increased the GFP signal in RecA-deficient *E. coli* (Figure 5c and d). The success in the rescue of RecA-deficient *E. coli* HR by RecAG204S suggested that the RecA-saturated nucleofilaments are dispensable for *in vivo* HR. Essentially, both RecAG204S and RecAS69G mainly formed the lightly assembled nucleofilaments *in vitro* (Figure 5b), and in turn, they could mediate strand exchange *in vitro* (Supplementary Figure S15) and partially rescue the HR pathway in RecA-deficient strains (Figure 5c and d). On the other hand, both ATP hydrolysis-deficient RecAK72R and RecAE96D mutants (Figure 5a), which favor RecA-saturated nucleofilaments (Figure 5b), failed to elicit homology-directed repair *in vivo* (Figure 5c and d). These results provide indirect evidence that the lightly assembled nucleofilaments, rather than the saturated nucleofilaments, mediate *in vivo* HR (Figure 5e).

Of note, to avoid the potential complication caused by the other effects, for example, SOS induction [32], the expression of all recombinant RecA proteins in the tested strains was controlled under the Lac Operon. The protein expression was further validated using western blotting analysis (Supplementary Figure S14B (c)).

### The binding of RecA to DNA is tightly regulated to facilitate *in vivo* HR

**Figure 6:**
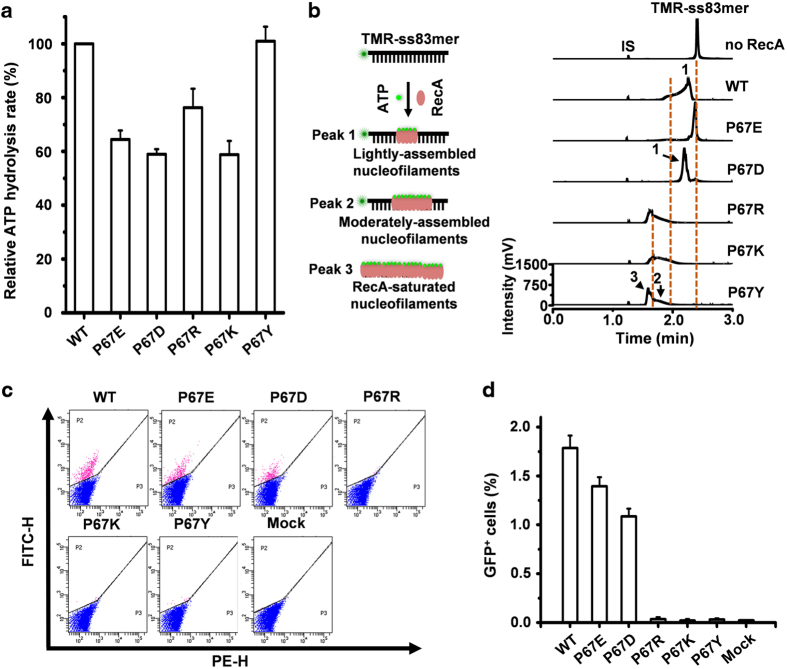
RecA mutations at P67 favoring the RecA-saturated nucleofilaments fail to mediate HR *in vivo* and those favoring the lightly assembled nucleofilaments can efficiently mediate HR *in vivo*. (**a**) ATP hydrolytic activity of five P67-mutated RecA proteins. (**b**) CE-LIFP electropherograms of the P67-mutated RecA-ssDNA filaments when stimulated by physiologically relevant ATP concentration (1.0 mM). The reactions also contained 10.0 nM TMR-ss83mer, 3.0 μM P67-mutated RecA protein as indicated in the figure, and proceeded at 37 °C for 10 min. Peaks 1–3 represent the lightly assembled, moderately assembled and saturated nucleofilaments, respectively. IS indicates the internal migration marker. (**c**, **d**) FACS analysis of *E. coli* HR efficiency of five RecA P67-mutants (**c**) and the percentage of GFP^+^ cells (**d**). FITC-H and PE-H (**c**) indicate the GFP signal and background fluorescence of the sorted *E. coli* cells, respectively. Pink and blue dots indicate GFP^+^ and GFP^−^ cells, respectively.

As described above, the formation of the lightly assembled nucleofilaments requires the ATP hydrolytic activity of RecA, suggesting a coupling of the RecA ATPase activity to the formation of the lightly assembled nucleofilaments. To further provide mechanistic insights into *in vivo E. coli* HR, we sought to uncouple the ATP hydrolytic activity from the regulation of the lightly assembled nucleofilament formation. As previously reported, ATP hydrolysis is determined by the highly conserved Walker A motif (spanning residues 66–73, GPESSGKT, in *E. coli* RecA). Among this motif, G66, G71, K72 and T73 are conserved and the other four residues are variable [33]. We therefore tested the substitution of one variable residue (P67) of RecA using one hydrophobic (Y), two acidic (D and E) and two basic (K and R) amino acid residues. *In vitro* assays showed that all these P67-mutated RecA proteins displayed efficient ATP hydrolytic activity (Figure 6a), however, only P67D and P67E formed the lightly assembled nucleofilaments (even with less density compared with wild type of RecA), but RecAP67Y/K/R formed a mixture of the moderately assembled and RecA-saturated nucleofilaments when they were stimulated by a physiologically relevant concentration of ATP (Figure 6b). Interestingly, only P67D and P67E stimulated *in vivo* HR (Figure 6c and d), although all the mutated proteins were highly expressed *in vivo* (Supplementary Figure S16). In addition, *recA*^*−/−*^ bacteria transfected with RecAP67D or RecAP67E showed correct *in vivo* HR repair as revealed by the fluorescence imaging of the reported cells (Supplementary Figure S17). All the *in vivo* HR data (Figures 5 and 6) are consistent with the supposition that RecA mutants favoring the lightly assembled nucleofilaments are compatible with *in vivo* HR.

## Discussion

### RecA-unsaturated nucleofilaments predominate and are tightly regulated by full ATPase activity

In this work, utilizing a novel CE-LIFP approach, we showed that ATP binding without subsequent hydrolysis, such as the use of the non-hydrolyzable ATP analog ATPγS, solely stimulates the formation of the RecA-saturated nucleofilaments. Similarly, those RecA mutants that retain ATP binding capability but lose ATP hydrolytic activity also tend to cooperatively assemble on ssDNA to form the saturated nucleofilaments. This is consistent with the literature [15, 21]. However, when ATP hydrolysis was included, we observed a consistent pattern of RecA binding to short oligos, for example, 1–4 monomers for polyT_12_, 1–5 monomers for polyT_15_ and 1–6 monomers for polyT_18_. Moreover, we could observe three types of RecA nucleofilaments for longer oligonucleotides, including lightly assembled, moderately assembled and RecA-saturated nucleofilaments. We found that the lightly assembled nucleofilaments are major components when stimulated by physiologically relevant ATP concentrations. In contrast, under the same conditions, the RecA-saturated nucleofilaments only accounted for less than 5% of the total nucleofilament population. Indeed, under physiologically relevant conditions, the RecA-unsaturated nucleofilaments predominate no matter how long the tested DNA, oligonucleotides (T18, ss83mer, ss175mer and circular ssDNA (5.6 K nucleotides). Even by increasing the concentration of RecA to 3–6 fold that of the physiologically relevant level, we mainly observed the moderately assembled nucleofilaments rather than the RecA-saturated nucleofilaments. In addition, the nuclease protection assay and EM validated the predominance of the lightly assembled nucleofilaments under physiological conditions.

**Figure 7:**
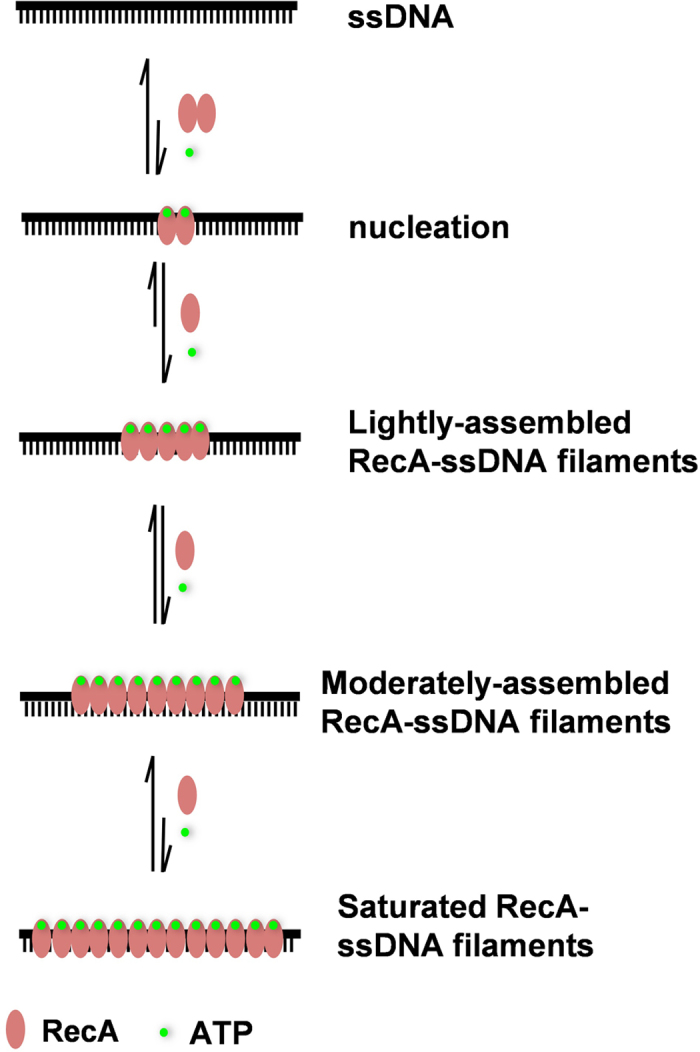
Dynamic model for the formation of diverse RecA nucleofilaments as stimulated by physiologically relevant ATP. First, two RecA monomers cooperatively nucleate on ssDNA; second, the nucleated RecA dimer further extends by assembling one RecA monomer on ssDNA each step to form the lightly assembled filaments; third, by same mechanism extends to form the moderately assembled filaments; and fourth, finally extends to form the saturated filaments. Due to tight regulation of the filaments by the RecA ATPase hydrolytic activity, the longer filaments will have more ATP hydrolysis and cause significant RecA dissociation, thereby, the unsaturated filaments (the lightly assembled filaments and the moderately assembled filaments) predominate and the saturated filaments are minor.

On the basis of the results obtained from CE migration, fluorescence polarization assay and nuclease protection assay, we proposed a biochemical model for the formation of dynamic and diverse RecA-ssDNA filaments (Figure 7). In this model, two RecA monomers first cooperatively nucleate on ssDNA, and extends by assembling one RecA monomer on ssDNA each step to form the lightly assembled filaments, the moderately assembled filaments and the saturated filaments. Because of the increase in the event number of ATP hydrolysis along with the number of RecA monomer in the filaments, the longer filaments should have more ATP hydrolysis, which leads to more RecA dissociation, however, the extension rate should be constant. When the RecA dissociation rate is equal to the extension rate, a dynamic equilibrium reaches. Finally, the unsaturated RecA-ssDNA filaments predominate in solution.

### RecA-unsaturated nucleofilaments function as a key scaffold for homologous recombination

Although the RecA-unsaturated nucleofilaments predominate under physiologically relevant conditions, we could observe minor RecA-saturated nucleofilaments (less than 5%). Therefore, we could not exclude the possibility that the minor RecA-saturated nucleofilaments rather than the major RecA-unsaturated nucleofilaments may be the critical structures to mediate homology recognition and strand exchange. To judge this possibility, we exploited a series of RecA mutants to investigate HR *in vitro* and *in vivo*.

Importantly, the RecA mutants (P67D and P67E) that have weakened DNA binding ability, but retain ATP hydrolytic activity, only form the RecA-unsaturated nucleofilaments and can mediate HR *in vitro* and *in vivo*. In contrast, all the tested RecA mutants (K72R, E96D, P67K, P67R and P67Y) favoring RecA-saturated nucleofilaments fail to mediate HR *in vivo*. In particular, some RecA mutants (P67K, P67R and P67Y) have efficient ATP hydrolytic activity, but they lose the ability to couple the ATP hydrolytic activity with the action to disassembly RecA monomers from the nucleofilaments to form less saturated nucleofilaments. These mutants also fail to mediate HR *in vivo*. These observations clearly support the perception that the RecA nucleofilaments are tightly regulated by ATPase activity, including both ATP binding and ATP hydrolysis, to remove excessively assembled RecA on ssDNA gap. This tight regulation is critical to the success of HR *in vivo*.

Interestingly, recent work [4] showed that a Rad51 paralog complex, RFS-1/RIP-1, can remodel presynaptic Rad51-ssDNA filaments to form an open and flexible conformation, as revealed by the sensitivity to nuclease DNase I digestion. By this mechanism, RFS-1/RIP-1 promotes Rad51-mediated homologous recombination in *Caenorhabditis elegans*. In our independent study, the identified active RecA nucleofilaments are also very sensitive to nuclease digestion. However, this is not simply related to the conformational change. The CE-LIFP assay enabled us to quantify binding stoichiometry, and as a result we were able to attribute this nuclease sensitivity to the formation of the lightly assembled nucleofilaments, thereby exposing most of nucleotides in the filaments to the nuclease. Of note, the formation of open and flexible Rad51 filaments in *Caenorhabditis elegans* requires the involvement of its paralog complex, but the formation of the active RecA nucleofilaments promoting HR does not require any paralog. Instead, RecA utilizes its intrinsic and DNA binding-dependent ATPase activity to remodel the nucleofilaments to facilitate HR.

In summary, by taking advantage of advanced CE-LIFP analysis, we have discovered that the physiologically relevant RecA nucleofilaments contain mainly RecA-unbound nucleotide sites. This observation was confirmed by nuclease protection assay and EM imaging. We further demonstrated that these novel RecA nucleofilaments coupled with ATP hydrolysis function as key scaffolds for homologous recombination.

## Materials and Methods

### Chemicals and reagents

The oligodeoxynucleotides were synthesized and purified by Shanghai Sangon Biological Engineering Technology & Services Co, Ltd (Shanghai, China). ATP, ATPγS (adenosine 5′-(γ-thio) triphosphate tetralithium), NADH (β-nicotinamide adenine dinucleotide, reduced disodium salt), and pyruvate kinase/lactic dehydrogenase were supplied by Sigma-Aldrich (St Louis, MO, USA). Phosphoenolpyruvate was purchased from Ruibio-bio (Ingelheim, Germany). Wild type RecA protein (*E. coli*) was purchased from New England Biolabs (Ipswich, MA, USA) or expressed and purified in our lab. All RecA mutants were expressed and purified in our own lab. Supernuclease was obtained from Sino Biological Inc. (Beijing, China). Ultrapure water of 18.2 MΩ cm was obtained from a Purelab Ultra Bioscience system (ELGA, UK). All other reagents and solvents were analytical grade and supplied by Beijing Chemical Reagents (Beijing, China).

### CE-LIFP

The CE-LIFP analyses were conducted on laboratory-built systems (Supplementary Figure S1A) [34]. Uncoated fused-silica capillaries with a dimension of 25 μm i.d. ×26 cm, 365 μm o.d. were used. Aliquots of the reaction solutions were electrokinetically injected into the capillary by applying an injection voltage of 15 kV for 5 s. Separation was carried out at room temperature with a voltage of 20 kV and an optimized buffer containing 25 mM Tris, 192 mM glycine, 0.5 mM Ca^2+^, pH 8.3. The additive Ca^2+^ was used to inhibit disassembly and stabilize the formed RecA nucleofilaments [18]. The TMR-labeled probes were excited at 543.5 nm, and the fluorescence was detected at 575 nm. The polarization (P) of the fluorescence was determined by measuring vertically and horizontally polarized fluorescence. The details are fully described in Extended Experimental Procedures.

### RecA assembly reactions

*E. coli* wild type or mutant RecA protein (0–3.0 μM) was mixed with a single-stranded oligonucleotide (10 nM, estimated as whole oligomer molecule) and one nucleotide cofactor unless otherwise stated. The RecA assembly solutions were prepared using a buffer of 20 mM Tris-HCl pH7.4, 10 mM Mg^2+^ (1×TH buffer). After 10 min incubation at 37 °C, the solutions were immediately subjected to CE-LIFP analysis or supernuclease assay.

### DNA strand exchange reactions

To perform strand exchange reactions, in general, RecA (3.0 μM), ssDNA (10 nM) and ATP (1.0 mM)/ATPγS (0.1 mM) were mixed in 1×TH buffer and incubated at 37 °C for 10 min, and then donor dsDNA (10 nM) was added to initiate the reactions. After incubation at 37 °C for an additional 10 or 20 min, the samples were immediately subjected to CE-LIFP analysis. The detail was described in Extended Experimental Procedures.

### Supernuclease assay

To confirm and test the assembly structures of RecA-ssDNA filaments, a nuclease assay was developed using supernuclease. Supernuclease is an extracellular non-specific endonuclease from *Serratia marcescens* [35–37], which hydrolyzes DNA and RNA in both single- and double-stranded forms at similar rates [38]. The conditions of RecA assembly were the same as that for CE-LIFP analysis except that ssDNA was labeled with Cy5 dye and the concentration (chains concentration) of ssDNA was raised to 20.0 nM for better gel imaging. After that, 5.0 units supernuclease was added and incubated at 37 °C for the designated times. Then, EDTA (100.0 mM final concentration) was applied to terminate the reactions, and the samples were further treated with 1.0 μg ml^−1^ protease K at 55 °C for 30 min. Finally, the samples were heated at 95 °C for 5 min, and analyzed by urea-denaturing PAGE (20%). The resultant gels were imaged using an infrared imaging system (Odyssey CLX, LI-COR Biotechnology, Lincoln, NE, USA).

### Generation of RecA mutants by site-directed mutagenesis

We generated pET21a-RecAs plasmids, containing the *recA*-mutated (G204S, E96D, S69G, P67E, P67D, P67R, P67K and P67Y) genes, by PCR-based site-directed mutagenesis using the Fast site-directed mutagenesis kit (Tiangen Biotech (Beijing) Co, Ltd, Beijing, China) according to the manufacturer’s instructions. pET21a-wtRecA, carrying wild-type *recA* allele inserted between the NdeI and HindIII sites, was used as the template for the pET21a-RecAs construction. Then, the constructed pET21a-RecAs was amplified and transformed into *E. coli* BLR(DE3) (Δ(srl-recA)) (Merck Millipore, Darmstadt, Germany) for protein expression and purification. All the primers involved were listed in Supplementary Table S2.

### Expression and purification of RecA mutants

RecAK72R was expressed and purified from *E. coli* STL2669.pT7 pol26 /pEAW481, carrying *recAK72R*, which was a kind gift from Michael M. Cox at the University of Wisconsin-Madison. The strains were cultured at 37 °C in LB containing 100 μg ml^−1^ ampicillin until A_600_ reached about 0.6. Then the culture was induced at 37 °C with 0.2 mM isopropyl-1-thio-β-D-galactopyranoside (IPTG) for 3 h. The proteins were purified according to the previous protocol with minor modification [31, 39]. Briefly, cells were collected and lysed, followed by polyethyleneimine precipitation, ammonium sulfate extraction and precipitation. The resultant pellet was re-suspended in 35 ml R buffer (20 mM Tris-HCl, pH 7.5, 1 mM DTT, 0.1 mM EDTA, 10% glycerol) plus 1 M ammonium sulfate and loaded onto a phenyl-sepharose low sub 6 FF column. The column was washed with three column volumes (CV) of the same buffer. Then, a 10×CV gradient elution of ammonium sulfate from 1 to 0 M in the R buffer was applied for separation. The RecAK72R mutant protein was collected in the flowthrough fractions. After dialyzing against R buffer containing 100 mM NaCl twice, the collected protein was loaded onto a Mono Q 10/100 GL column, and washed with 5×CV of R buffer containing 100 mM NaCl. Then, the column was eluted with 15×CV gradient solution from R buffer plus 100 mM NaCl to R buffer plus 1.0 M NaCl. RecAK72R protein was eluted at about 400 mM NaCl. The fractions containing RecAK72R were pooled and dialyzed against R buffer containing 100 mM NaCl (with 25% glycerol) overnight. The purity of RecAK72R protein was identified by 12% SDS-PAGE, and concentration was determined with the Bradford assay using BSA standard. Finally, the purified protein was split and stored at −80 °C.

The wild type and other mutant RecA proteins were also expressed and purified using the same protocol as described above except RecAE96D. Of note, RecAE96D could be retained on phenyl-sepharose low sub 6 FF column and was only eluted by R buffer (without ammonium sulfate).

### *In vivo E. coli* homologous recombination assay

This is a modification of a previously described mammalian assay [40]. We exploited I-SceI, which is a rare-cutting restriction endonuclease, and two inactivated tandemly repeated green fluorescent protein (DR-GFP) genes (see Supplementary Figure S14A). One GFP gene copy (SceGFP) was inactivated by one I-SceI site, which was incorporated into a naturally occurring BcgI restriction site by substituting 11 bp of the wild-type gene. The incorporated I-SceI site contained two inframe stop codons terminating the translation of SceGFP. The other copy (iGFP) contained one BcgI site as a template for homology-directed repair of the I-SceI-cut SceGFP gene. The downstream iGFP was inactivated by a downstream truncation (218 bp). The correct GFP can only be generated through homology-driven repair. The detail was estimated as described in Extended Experimental Procedures.

### Fluorescence-activated cell sorter (FACS) analysis of recombination efficiency

The modified *E. coli* strains stably co-transformed with plasmids were cultured in 10 ml LB media with 10 μg ml^−1^ tetracycline at 37 °C until A_600_ reached 1.0. Then the strains were centrifuged at 4 000 rpm for 5 min at room temperature, and re-suspended in fresh 10 ml LB containing 10 μg ml^−1^ tetracycline and 0.4 mM IPTG, and further incubated at 37 °C for 8 h for generation of double-stranded breaks, homology-directed repair and GFP expression. LB media was replaced once with fresh 20 ml media containing 10 μg ml^−1^ tetracycline and 0.40 mM IPTG after 4 h incubation. After incubation, 1.0 ml cultures were centrifuged at 4 000 rpm for 5 min at 4 °C. The cell pellets were collected and washed with cold sterile PBS three times, finally suspended and kept in cold PBS. Then the strains were analyzed by FACS with FITC and PE channels. FITC channel (Ex: 488 nm; Em: 530 nm) was used for measuring GFP signal, and PE channel (Ex: 488 nm; Em: 585 nm) was for background fluorescence. The gate was set with a strain only carrying p15A-GFP plasmids. The efficiency of GFP positive (GFP^+^) cells was estimated as the ratio of GFP^+^ cells to the total cells analyzed.

### Electron microscopy (EM) analysis

EM analysis was carried out on a FEI transmission electron microscope. 3.0 μM RecA, nucleotide cofactor (1.0 mM ATP or 0.1 mM ATPγS), and 300.0 nM ss83mer were mixed in the RecA assembly buffer (1×TH buffer), and incubated at 37 °C for 10 min. For RecA controls, no ss83mer was included. Then, the samples were diluted five-fold using 1×TH buffer with desired nucleotide cofactor (1.0 mM ATP or 0.1 mM ATPγS). A total of 3.0 μl diluted solution for each sample was loaded onto a glow-discharged copper grid coated with carbon film for 40 s followed by conventional negative stain with 2% uranyl acetate. Images were collected by the TEM operated at 200 kV and recorded with a Ceta 4096×4096 pixel CCD camera (FEI).
