# Supplementary material for: ATPase activity tightly regulates RecA nucleofilaments to promote homologous recombination
Source: Cell Discov. 2017 Jan 17;3:16053–. doi: 10.1038/celldisc.2016.53 (PMC5240526; doi:10.1038/celldisc.2016.53)
Supplement: Supplementary Information [file celldisc201653-s1.pdf]

Supplementary Information

ATPase activity tightly regulates RecA nucleofilaments to promote homologous recombination

Supplementary Information includes Extended Experimental Procedures, Seventeen Supplementary Figures, and Two Supplementary Tables, all of which can be found with this article online.

Extended Experimental Procedures

Capillary electrophoresis and laser-induced fluorescence polarization (CE-LIFP) analysis.

CE-LIFP analysis was performed on a laboratory-built instrument as described previously[1]. The setup is illustrated in Supplementary Figure S1. It mainly consists of two coupled systems, the capillary separation system (Supplementary Figure S1A) and the fluorescence excitation and detection system (Supplementary Figure S1B). For the excitation and detection system, a helium-neon laser (1.0 mW, 543.5 nm, vertically polarized light, Melles Griot, Irvine, CA) was used to excite fluorescent molecules. The emitted fluorescence was split into horizontally and vertically polarized light by a polarizing beam splitter (Melles Griot, Nepean, Canada), which were detected by two photomultiplier tubes (PMT, model R1477, Hamamatsu Photonics, Japan) at 575 nm, respectively. For the capillary electrophoresis separation system, uncoated fused-silica capillaries (Yongnian Optic Fiber Plant, Hebei, China) were used with a total length of 26 cm, effective separation length of 20 cm with detection window at position of 20 cm away from the inlet, and dimensions of 25 μm i.d. × 365 μm o.d. unless otherwise specified. Samples were electrokinetically injected into the capillary by applying a voltage of 15 kV for 5 s and separated by a voltage of 20 kV at room temperature. Samples were prepared in 1 × TH buffer (20 mM Tris-HCl buffer, pH 7.4, 10 mM Mg^{2+}) unless otherwise stated. A running buffer comprising of 1 × TG pH8.3 (25mM Tris, 192mM glycine) plus 0.5 mM Ca^{2+} was used for CE separation. In this case, Ca^{2+} functioned as an additive of CE separation. Essentially, Ca^{2+} prevented the dissociation of the formed RecA-ssDNA filaments[1], facilitating accurate characterization of assembly stoichiometry of RecA on ssDNA.

The fluorescence polarization (P) values were calculated from the obtained intensities of the horizontally polarized fluorescence (I_h) and vertically polarized fluorescence (I_v) according to the following equation (Supplementary Figure S1C):

$$P = \frac{I_v - I_h}{I_v + I_h}$$

DNA strand exchange reactions.

To perform strand exchange reactions, ssDNA, ATP/ATPγS, and RecA were sequentially added and mixed in the 1 × TH buffer (20 mM Tris-HCl buffer, pH 7.4, 10 mM Mg^{2+}) and incubated for 10 min at 37 °C, and then donor dsDNA was added to initiate the reactions. After incubation at 37 °C for an additional 10 or 20 min, the samples were immediately subjected to CE-LIFP analysis or native PAGE analysis with 1×TBE buffer. The concentrations of oligonucleotides were all shown in terms of oligonucleotide chains. The sequences and labeling of oligonucleotides were described in Supplementary Tables S1 and S2 and also in the figure legends.

To facilitate the gel imaging, in PAGE analysis of strand exchange products, the DNA probes were fluorescently labeled by Cy5 dye at the 5’ end. Other conditions were stated in the figure legends.

Construction of *in vivo E. coli* recombination system.

The GFP-reporter system for *in vivo E.coli* recombination assay was constructed by modifying the mammalian DR-GFP system developed in Dr. M. Jasin’s lab[2]. The constructed *E. coli* reporter system contained three types of compatible plasmids p15A-GFP, pSC-I-SceI and pET21a-RecAs. p15A-GFP and pSC-I-SceI carried

DR-GFP and I-SceI (rare-cutting endonuclease) genes, respectively. pET21a-RecAs contained wild-type RecA or mutated RecA. All the target genes are expressed under the control of the T7 promoter.

p15A-GFP was constructed from the base of pACYC184 (ATCC 37033) carrying p15A ori. T7 promoter and lac operator were first inserted into pACYC184 using EagI and HindIII sites, forming p15A-T7 plasmid. The DNA fragment containing both T7 promoter and lac operator was amplified using the sequence between SphI and NdeI sites of pET21a(+) (Novagen®) as template. The primers for PCR of the fragment containing both T7 promoter and lac operator were as below:

Forward primer: 5'- ATACGCCGGCCGGCATGCAAGGAGATGGCGCCCAACA
GTCC-3'

Reverse primer: 5'- ATACGCAAGCTTGAGCTCCCGCCGGGTACCTATATCTCC
TTCTTAAAGTTAAACAAAATTATTTCTAGAGG-3'.

The above primers carried EagI and HindIII sites for insertion of T7 promoter and lac operator into pACYC184. They also contained KpnI and SacI sites, which were used for DR-GFP insertion. Then, the DR-GFP gene was amplified from the pDR-GFP plasmid and ligated into the constructed p15A-T7 plasmid between KpnI and SacI sites. The primers for amplification of DR-GFP from pDR-GFP plasmid are shown below:

Forward primer: 5'-ATACGCGGTACCATGCATTTTGGCAAAGAATTCAGAT
CCGCCGCCAC-3'

Reverse primer: 5'-ATACGCGAGCTCCAGCTATGACCATGATTACGCCAAGC
TTTTATTG-3'.

The pSC101-I-SceI was constructed by sequential ligation of four fragments containing distinct functional elements. The first fragment, carrying pSC101 ori and Rep101, was amplified from pKD46 plasmid (Tiandz, Inc., Beijing, China) with primers:

Forward primer: 5'- ATACGCCTCGTGGGTGCCTCACTGATTAAGCATTGG-3'

Reverse primer: 5'-ATACGCTGTACAACGAGTATCGAGATGGCACATAGCC-3'

The second fragment carrying Kan resistance gene was amplified from pET28a(+) (Novagen®) with the primers:

Forward primer: 5'-ATACGCCACGAGGCCGCCGGTACCGAAGATCCTTTG ATCTTTTCTACGGGGTCTGACGC-3'

Reverse primer: 5'-ATACGCTGTACACAGGTGGCACTTTTCGGGGAAATGT GCGC-3'.

Then, the above two fragments were ligated into a circular DNA through BssSI and BsrGI sites, which was marked as pSC101-Kan plasmid.

The third fragment containing both T7 promoter and lac operator amplified from pET21a(+) was inserted into the region between BssSI and KpnI sites of the constructed pSC101-Kan plasmid, forming pSC101-T7 plasmid.

Finally, the fourth fragment, carrying I-SceI gene from pCβASce[2], was inserted into the pSC101-T7 plasmid with NdeI and SacI sites, forming pSC101-I-SceI plasmid. The primers for T7 promoter and I-SceI amplification were:

Forward primer: 5'-ATACGCGGTACCGCGCCATTCGATGGTGTCCGGGATC-3'

Reverse primer: 5'-ATACGCCACGAGCCGGATATAGTTCCTCCTTTCAGC-3'

The primers for I-SceI amplification were:

Forward primer: 5'- ATACGCCATATGTTCATGCCTTCTTCTTTTTCCTACAG

C-3’

Reverse primer: 5’- ATACGCGAGCTCCCTTCTGATAGGCAGCCTGCACCTG AGGAG-3’

The pET21a-wtRecA and pET21a-mutated RecA were constructed with wild-type *recA* and mutated *recA* inserted between the NdeI and HindIII sites in pET21a(+). The pET21a(+) plasmid without containing any *recA* gene was used as a mock control. The target genes were confirmed by DNA sequencing.

The constructed p15A-GFP was first transformed into *E.coli* BLR(DE3) (Δ(srl-recA)) competent cells (Merck Millipore, Germany) using heat shock strategy. A positive clone was selected based on PCR assay. Then the positive clone was amplified and made competent using $CaCl_2$, followed by the transformation of the next plasmid, pET21a-RecA (wtRecA or RecA mutants or mock plasmid). Similarly, the pSC101-I-SceI plasmid was transformed into the competent strains containing both p15A-GFP and pET21a-RecA. The presence of each plasmid in the strains was confirmed by PCR assay. The primers were designed as below:

GFP-F: 5’-ATCCCTCGACCTGCAGCCCAAGCTTTAGG-3’

GFP-R: ’-GAGCTCCAGCTATGACCATGATTACGCCAAGCTTTTATTG-3’

I-SceI-F: 5’-GAGCTCCCTTCTGATAGGCAGCCTGCACCTGAGGAG-3’

I-SceI-R: 5’-CATATGTTCATGCCTTCTTCTTTTTCCTACAGC-3’

RecA-F: 5’-TTAATGCGCCGCTACAGGGCGCGTCC-3’

RecA-R: 5’-GACTCTCTTCCGGGCGCTATCATGCC-3’

The sizes of PCR products were about 0.85 Kb, 1.0 Kb and 1.7 Kb for plasmids

containing GFP, I-SceI and RecA, respectively.

Western blot for RecA protein expression

E.coli strains carrying functional plasmids were centrifuged after IPTG induction and washed twice with PBS, then the cell pellets were lysed with Bugbuster reagent (Novagen). The total protein concentrations were measured using the Bradford Assay, and 10.0 μg total protein each was separated on an 8% SDS-PAGE. Then proteins were transferred to a nitrocellulose membrane, blocked with 5% non-fat milk and incubated overnight at 4 ˚C with antibody against RecA (Abcam, 1:300). Blots were incubated with secondary antibody against rabbit (1: 6000) for 1h before visualization.

REFERENCES

1. Wang H, Lu M, Tang MS, Van Houten B, Ross JB, Weinfeld M, Le XC. DNA wrapping is required for DNA damage recognition in the *Escherichia coli* DNA nucleotide excision repair pathway. *Proc. Natl. Acad. Sci. USA* 2009; **106**: 12849-12854.
2. Pierce AJ, Johnson RD, Thompson LH, Jasin M. XRCC3 promotes homology-directed repair of DNA damage in mammalian cells, *Genes Dev.* 1999; **13**: 2633-2638.

Supplementary Tables

Supplementary Table S1. Sequences of DNA probes for RecA assembly and strand exchange.

Name	Sequence (5’→3’)
(+) ss83mer	TTT CCT ACC TTA AGA TCC TTC CAG TCT CCG CCG GCC AGT GTT ACC CTT AGA GCT CAT ACC ATT CGC CAA TTT CTT CGC ACG TT
(-) ss83mer	AAC GTG CGA AGA AAT TGG CGA ATG GTA TGA GCT CTA AGG GTA ACA CTG GCC GGC GGA GAC TGG AAG GAT CTT AAG GTA GGA AA
(+) 175mer	TCG TCT TTG GTA TGT AGG TGG TCA ACA ATT TTA ATT GCA GGG GCT TCG GCC CCT TAC TTG AGG ATA AAT TAT GTC TAA TAT TCA AAC TGG CGC CGA GCG TAT GCC GCA TGA CCT TTC CCA TCT TGG CTT CCT TGC TGG TCA GAT TGG TCG TCT TAT TAC CAT TTC AAC TAC TCC G
(-) 175mer	CGG AGT AGT TGA AAT GGT AAT AAG ACG ACC AAT CTG ACC AGC AAG GAA GCC AAG ATG GGA AAG GTC ATG CGG CAT ACG CTC GGC GCC AGT TTG AAT ATT AGA CAT AAT TTA TCC TCA AGT AAG GGG CCG AAG CCC CTG CAA TTA AAA TTG TTG ACC ACC TAC ATA CCA AAG ACG A
T_n	$\underbrace{\text{TTT TTT…TTT TTT}}_{n}$

Notes: 1. The fluorescence dye TMR was labeled at 5’-end for the indicated oligos, and Cy5 was labeled at 3’-end for the indicated oligos.

2. ds83mer was obtained by annealing (+)ss83mer and (-)ss83mer.

3. When fluorescence labeled dsDNA probes were used, the dye was labeled at 5’-end of (+) strand unless otherwise specified;

4. The (+) strand was used as incoming ssDNA for RecA assemblage.

Supplementary Table S2. Primers used for RecA mutagenesis

	Name	Sequence (5'→3')
G204S	Forward	AATTGGTGTGATGTTCAGTAACCCGGAAACCACTAC
	Reverse	TGAACATCACACCAATTTTCATACGGATCTGG
S69G	Forward	ATCTACGGACCGGAAGGCTCCGGTAAAACCACG
	Reverse	CGTGGTTTTACCGGAGCCTTCCGGTCCGTAGAT
E96D	Forward	GTGCGTTTATCGATGCTGATCACGCGCTGGACCC
	Reverse	ATCAGCATCGATAAACGCACAGGTTTTACCTTCACG
P67E	Forward	CGTCGAAATCTACGGAGAAGAATCTTCCGGTAAAACC
	Reverse	GGTTTTACCGGAAGATTCTTCTCCGTAGATTTCGACG
P67D	Forward	CGTCGAAATCTACGGAGATGAATCTTCCGGTAAAACC
	Reverse	GGTTTTACCGGAAGATTCATCTCCGTAGATTTCGACG
P67R	Forward	CGTCGAAATCTACGGACGCGAATCTTCCGGTAAAACC
	Reverse	GGTTTTACCGGAAGATTCGCGTCCGTAGATTTCGACG
P67K	Forward	CGTCGAAATCTACGGAAAAGAATCTTCCGGTAAAACC
	Reverse	GGTTTTACCGGAAGATTCTTTTCCGTAGATTTCGACG
P67Y	Forward	CGTCGAAATCTACGGATATGAATCTTCCGGTAAAACC
	Reverse	GGTTTTACCGGAAGATTCATATCCGTAGATTTCGACG

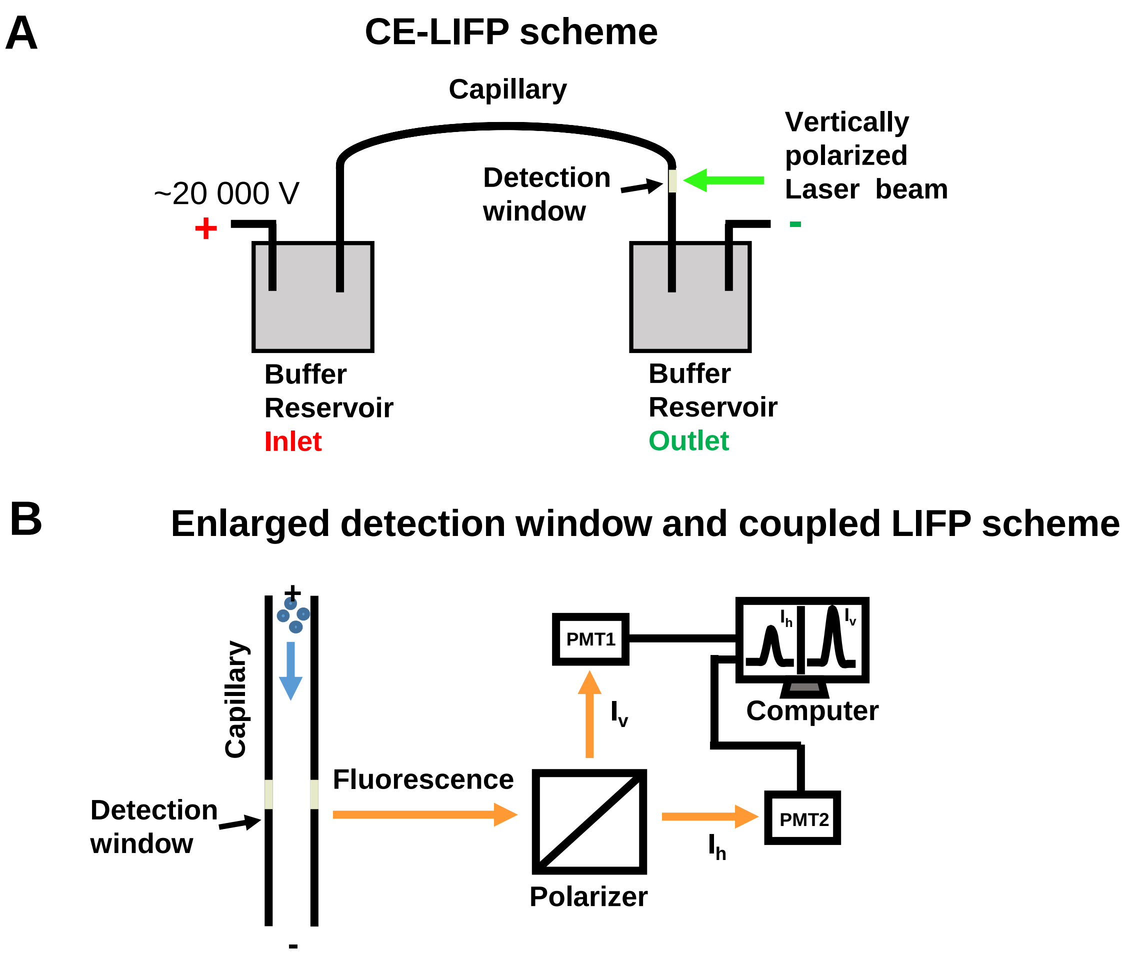

C

$$P = \frac{I_v - I_h}{I_v + I_h}$$

Supplementary Figure S1. CE-LIFP aparatus.

(**A**) Laboratory-built CE-LIFP setup. A voltage of ~20 kV is usually applied between the two ends of the fused silica capillary (inner diameter: ~25 μm; 26 cm long), which is filled only with pure separation buffer (no gel, no polymer). The protein-bound DNA and unbound DNA are efficiently separated according to their charge/mass ratio. In our system, protein-bound DNA migrates faster than unbound DNA due to a dilution of negative charge of DNA by the bound protein (approximately zero charge). A detection window is set near ground end. Vertically polarized laser beam is perpendicularly focused in the circle point of the capillary. The fluorescent species when passing the focal plane is excited by the intensively focused laser light and emit strong fluorescence, which can be sensitively detected by the photomultiplier tubes set in the dark bin (not shown).

(**B**) Schematic illustration of on-line coupled fluorescence polarization detection. The emitted fluorescence is often depolarized by the molecular rotation in the process of fluorescence emission. In theory, if the molecules are static and orients randomly, the maximum polarization response can be generated (P = 0.5), for example, immobilized fluorescence dye. If the molecules that rotate extremely fast at nanosecond scale would generate a polarization value of zero, for example, small fluorescence dyes (approximate to zero). To measure the polarization, the fluorescence light is split into vertically polarized light (I_v) and horizontally polarized light (I_h).

(**C**) The formula for quantitative description of fluorescence polarization (P, dimensionless).

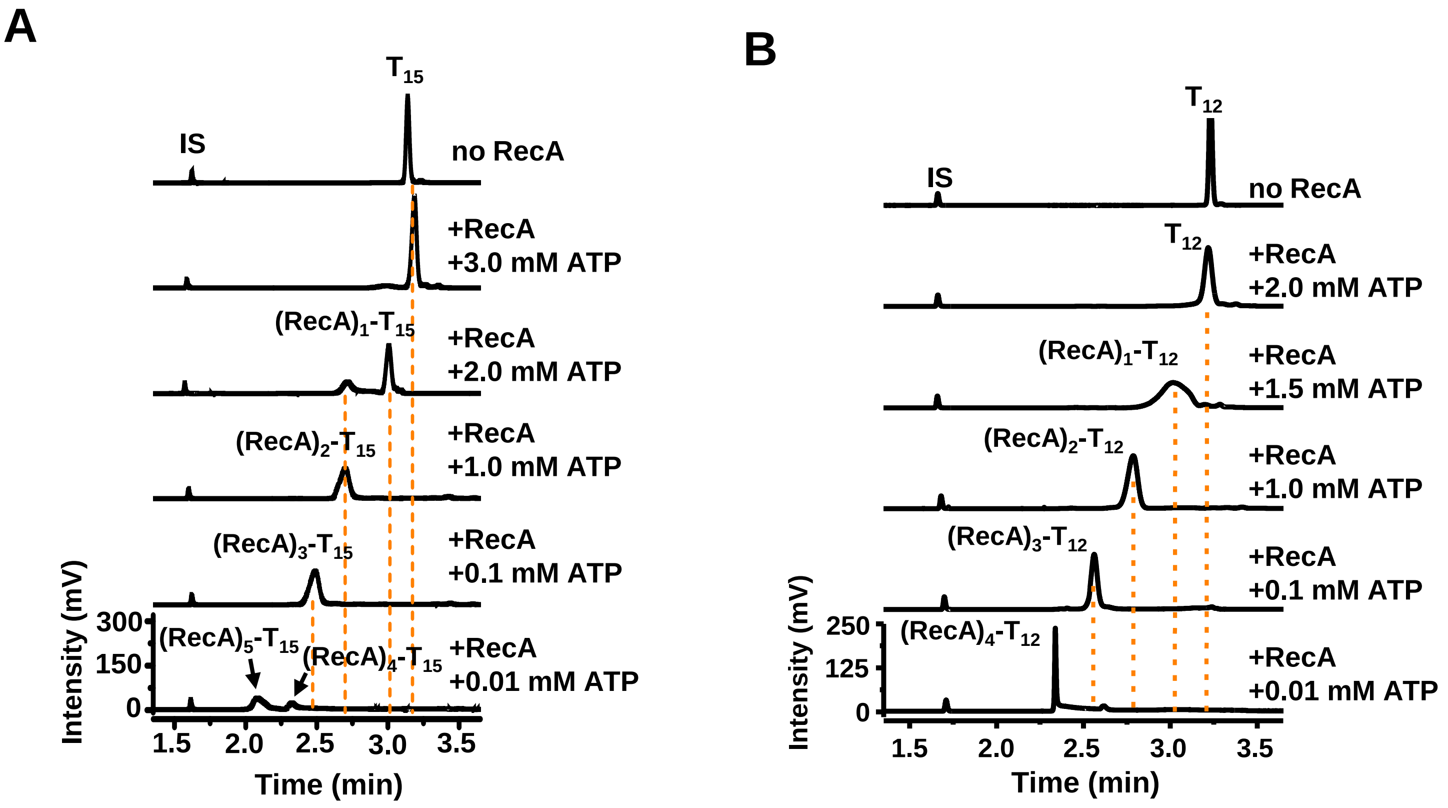

Supplementary Figure S2. The binding pattern for the assembly of RecA on short oligonucleotides as stimulated by ATP hydrolysis.

Electropherograms obtained from CE-LIF analysis of the mixtures of 10 nM single stranded oligo (TMR-T_{15} (**A**) or TMR-T_{12} (**B**)) and 16 μM RecA in the presence of ATP (0.01 -3.0 mM), showing the formation of five complexes for TMR-T_{15} (**A**) or four complexes for TMR-T_{12} (**B**).

Notice: In all CE-LIFP electropherograms, IS indicates an internal migration marker.

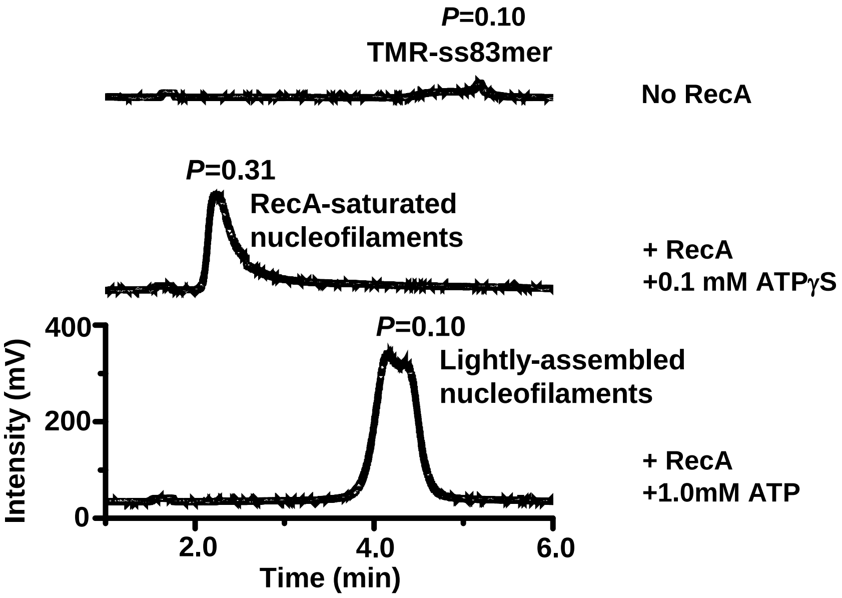

Supplementary Figure S3. The predominance of the lightly-assembled nucleofilaments was confirmed using a separation buffer containing ATP and Mg^{2+}.

The assembly of RecA on ssDNA involved with ATP and Mg^{2+}, and an optimized buffer containing 25mM Tris, 192 mM glycine, 0.5 mM Ca^{2+}, pH 8.3 was used for CE separation to preserve the RecA-saturated nucleofilaments. However, there is one possibility that the observed lightly-assembled nucleofilaments might be artificially detected due to the absence of ATP and Mg^{2+}, which were included in the reaction buffer but not in the separation buffer. To examine this possibility, a buffer of 20 mM Tris-HCl, pH 7.4, plus 0.5 mM ATP and 1.0 mM Mg^{2+} was used for CE separation here, and neither of the reaction and separation was involved with Ca^{2+} . As shown in this figure, the lightly-assembled nucleofilaments predominated even using the separation buffer containing ATP and Mg^{2+}.

Of note, the fluorescence of TMR-ss83mer was quenched in the presence of ATP + Mg^{2+} due to the collisions between the labeled fluorescent dye of TMR-ss83mer and ATP + Mg^{2+}, and thus only weak fluorescence signal was observed (see top trace). However, RecA binding could protect the fluorescent dye of TMR-ss83mer from such collisional quenching, and thus the fluorescence signal recovered (middle and bottom traces).

The ingredients included in each sample were indicated as in the figure.

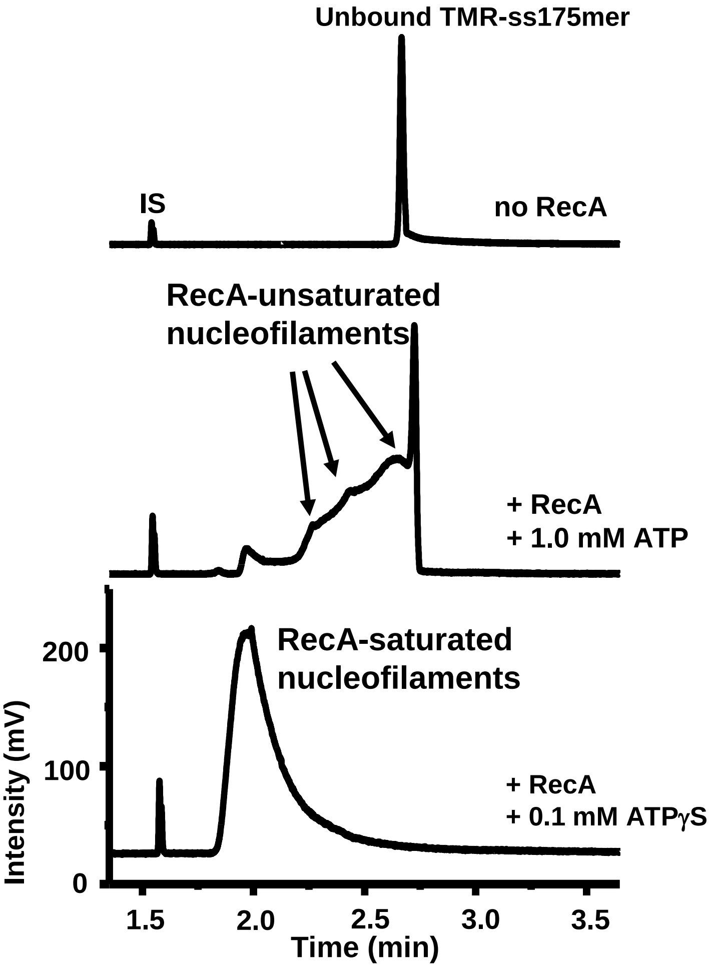

Supplementary Figure S4. The predominance of the RecA-unsaturated nucleofilaments for longer oligonucleotide (175mer) as stimulated by ATP hydrolysis

CE-LIFP electropherograms were obtained for analysis of RecA assembly on TMR-ss175mer.The reaction solution included 3.0 μM RecA,10 nM TMR-ss83mer, and one nucleotide cofactor (0.1 mM ATPγS or 1.0 mM ATP), and proceeded for 10 min at 37 °C.

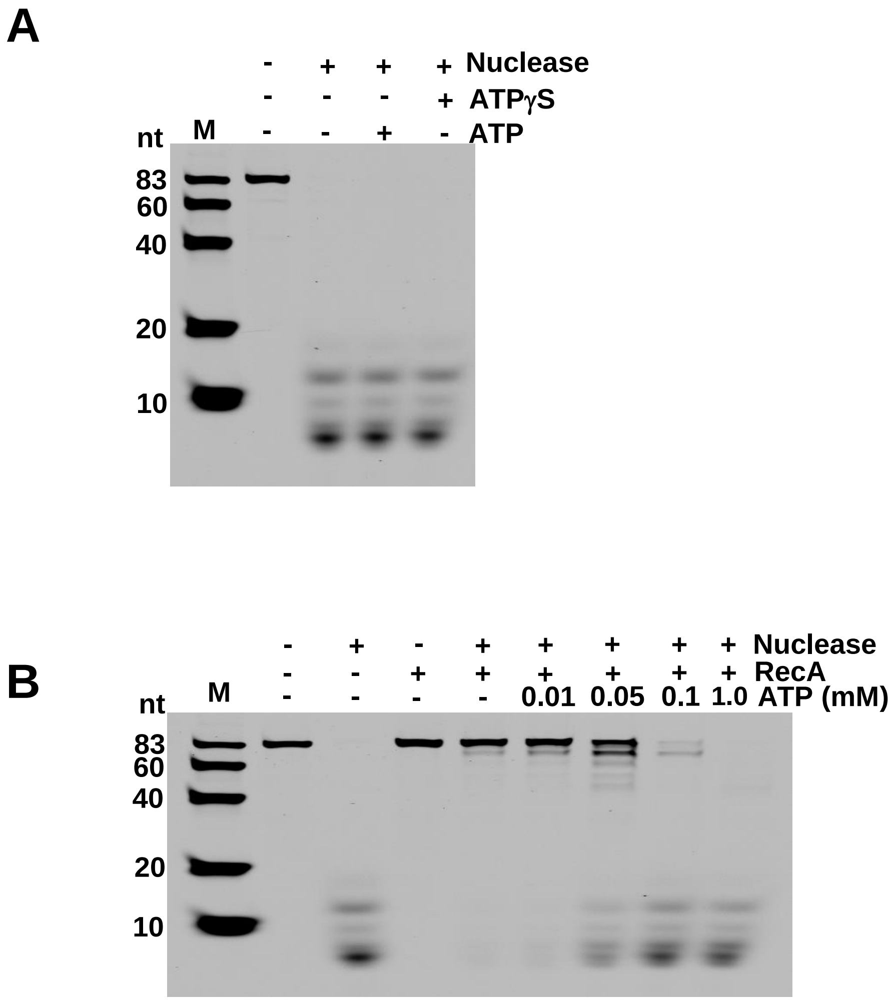

Supplementary Figure S5. The formation of the lightly-assembled nucleofilaments stimulated by ATP hydrolysis was confirmed by supernuclease assay.

(**A**) Nuclease digestion of ssDNA (Cy5-ss83mer) in the absence or presence of nucleotide cofactor.

(**B**) Nuclease digestion of the RecA-Cy5-ss83mer complexes stimulated by ATP of varying concentrations.

The reactions were terminated using 100 mM EDTA, and further treated with 1.0 μg/mL proteinase K at 55 °C for 30 min. Then, the samples were heated at 95 °C for 5 min, and analyzed by urea-denaturing polyacrylamide gel electrophoresis (20% PAGE). M, ssDNA markers (nts); Cy5 is a fluorescent label; Supernuclease: 5.0 units; Digestion time: 30 min; Cy5-ss83mer: 20 nM. (**A**) ATP: 1.0 mM; or ATPγS: 0.1 mM. (**B**), RecA: 3.0 μM.; ATP used at the concentrate as indicated.

A

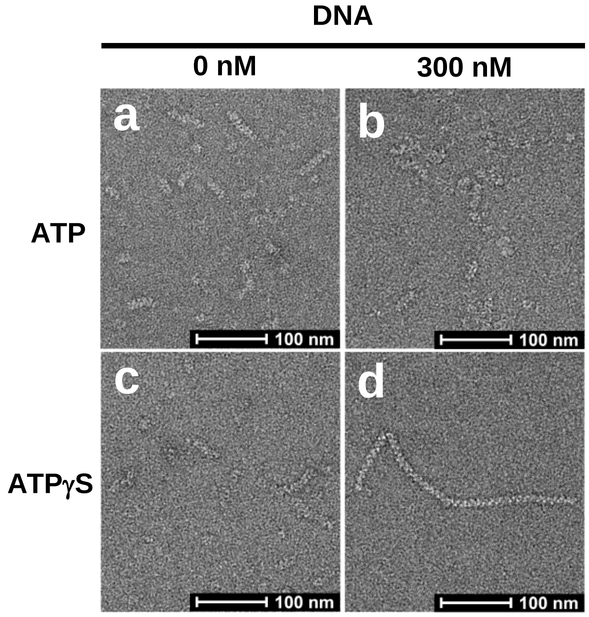

B

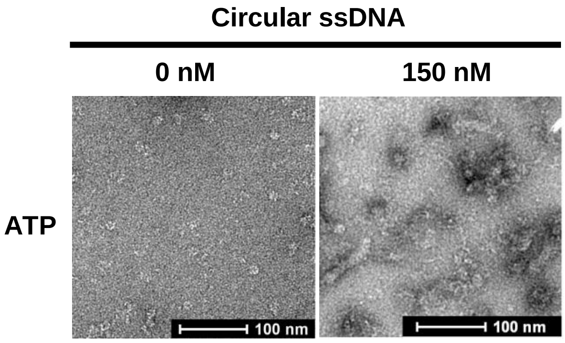

C

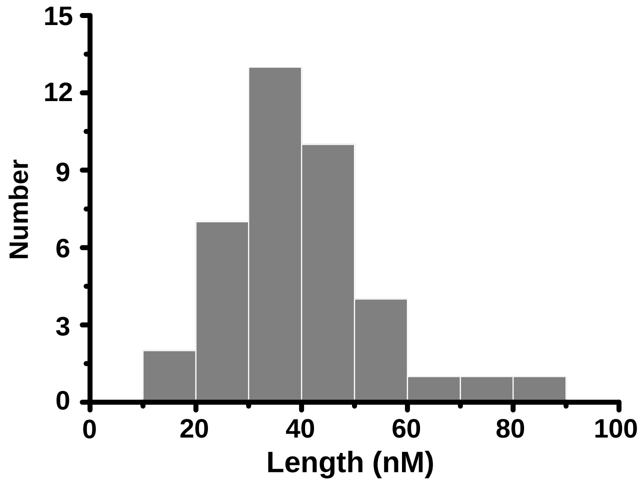

Supplementary Figure S6. EM imaging of the RecA-unsaturated nucleofilaments stimulated by ATP hydrolysis.

(A) EM analysis of RecA-ssDNA (ss83mer) filaments stimulated by ATP (1.0 mM, a, b) or ATPγS (0.1 mM, c, d) in the presence of RecA (3.0 μM). Note, we did not observe any ATP hydrolytic activity of pure RecA (data not shown), suggesting that no RNA was bound to the purified RecA. High concentration of ss83mer (300 nM) was used to eliminate the RecA filaments that did not contain any DNA.

(B) EM analysis of RecA-ssDNA(single strand DNA plasmid: ΦX174 virion DNA) filaments stimulated by ATP (1.0 mM) in the presence of RecA (3.0 μM). High concentration of ΦX174 virion DNA(150 nM) was used to eliminate the RecA filaments that did not contain any DNA.

(C) Statistic summary of the length of ΦX174 virion DNA-RecA nucleofilaments (n = 39) stimulated by ATP (1.0 mM) in the presence of RecA (3.0 μM).

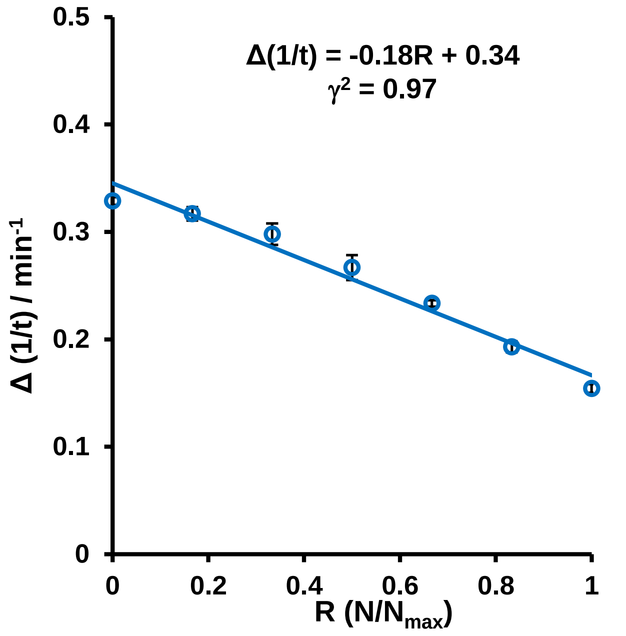

Supplementary Figure S7. Calibration for the binding stoichiometry of RecA and oligonucleotide.

Linear fit of Δ(1/t) of the RecA-ssDNA complexes against the ratio (R) of the bound RecA number (N) to the maximum bound number (N_{max}) per oligomer. Δ(1/t) = $1/t_0 – 1/t$, and t_0 and t represents the migration time of IS and the RecA-T_{18} complexes, respectively.

A

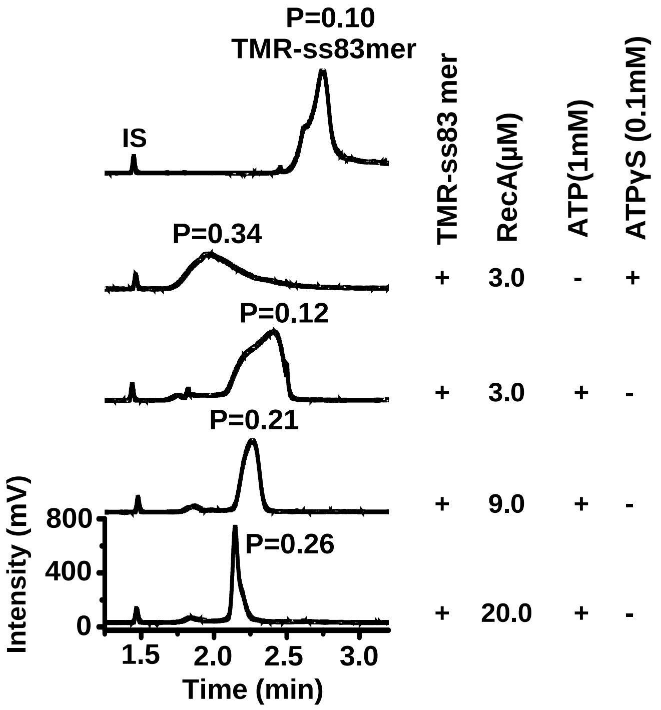

B

RecA concentration (μM)*	Bound number
3.0	~5.0
9.0	~15.0
20	~19.0

*ATP: 1.0 mM

C

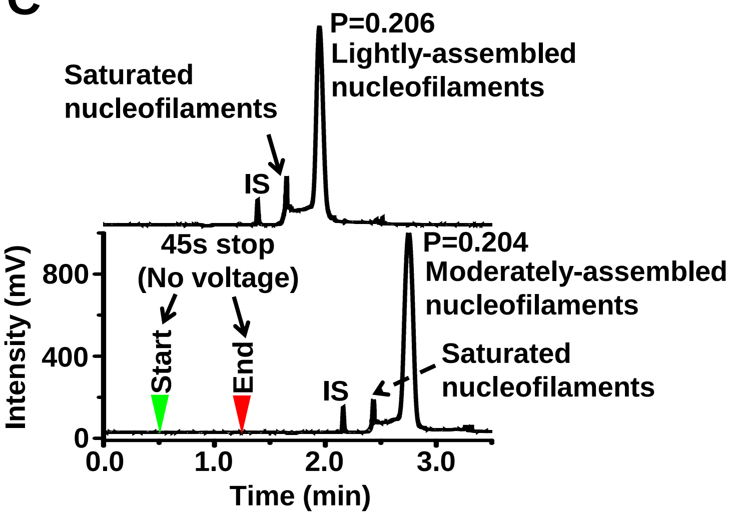

Supplementary Figure S8. The formation of the moderately-assembled nucleofilaments rather than RecA-saturated nucleofilaments in the presence of excess RecA.

(**A**) Electropherograms obtained from CE-LIFPanalysis of the mixtures of TMR-ss83mer (10 nM) and RecA at high concentration (up to 20 μM) in the presence of ATP (1.0 mM), showing the preferential formation of the moderately-assembled nucleofilaments rather than RecA-saturated nucleofilaments saturated nucleofilaments even involved with excess RecA.

(**B**) The estimated numbers of RecA binding to TMR-ss83mer for RecA of varying concentration (3.0 - 20 μM).

(**C**) Electropherograms obtained from CE-LIFP analysis of the moderately-assemled nucleofilaments with or without 45-second stop, showing the stability of the moderately-assemled nucleofilaments throughout CE separation. The reactions contained 9.0 μM RecA, 10 nM TMR-ss83mer, and 1.0 mM ATP, and proceeded at 37 °C for 10 min.

Note: the RecA stock solution (100 μM) contained 50 % glycerol, and thus the reactions for 20 μM (final) RecA involved with about 10% glycerol. In other reactions (involved with 3.0 or 9.0 μM RecA, Supplementary Figure 8A), additional glycerol was also included to maintain final 10% glycerol.

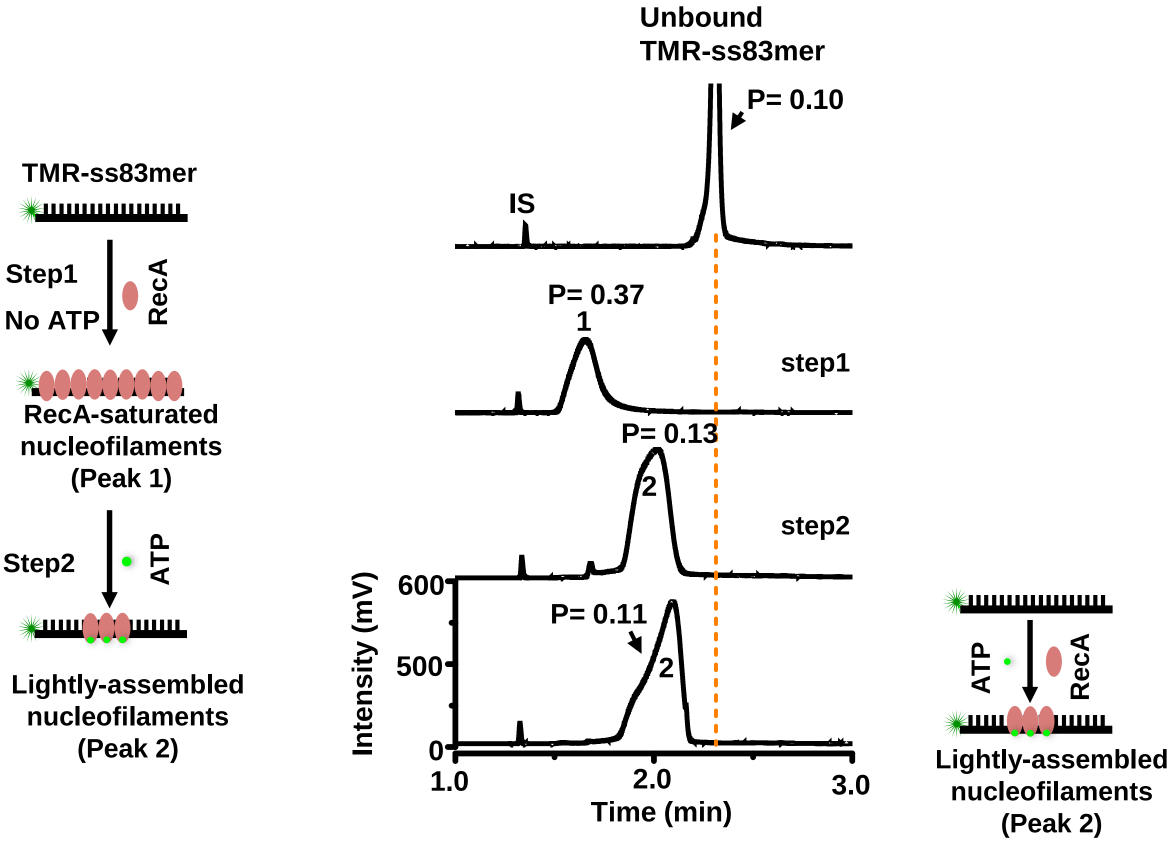

Supplementary Figure S9. ATP hydrolysis promotes the formation of the lightly-assembled RecA nucleofilaments.

Electropherograms obtained from CE-LIFP analysis of the mixture of RecA (3.0 μM) and TMR-ss83mer (10 nM) before (Step 1) and after (step 2) addition of ATP (1.0 mM), showing that RecA-saturated nucleofilaments are instable and converted to the lightly-assembled RecA nucleofilaments when stimulated by ATP. By directly mixing RecA, ATP and ssDNA (bottom trace), only the lightly-assembled nucleofilaments were observed (peak 2, bottom trace).

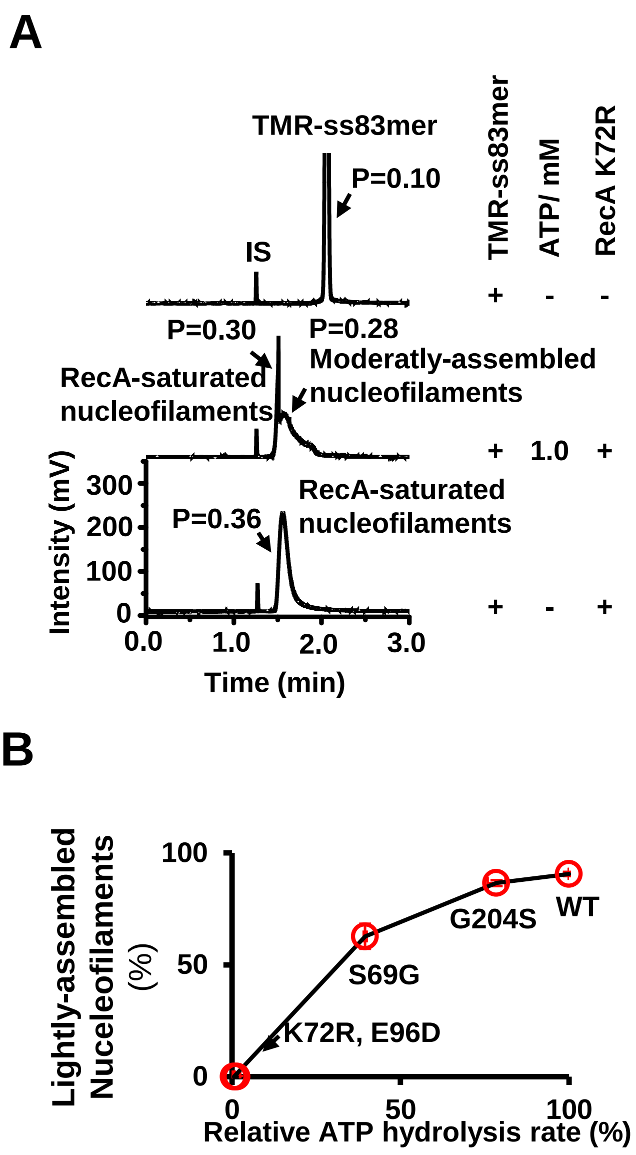

Supplementary Figure S10. ATP hydrolytic activity is required for the formation of the lightly-assembled RecA nucleofilaments.

(**A**) The mutant protein RecA K72R can bind ATP but cannot hydrolyze ATP, and thus fails to form the lightly-assembled RecA nucleofilaments.

(**B**) The correction of ATP hydrolysis rate with the abundance of the lightly-assembled RecA nucleofilaments.

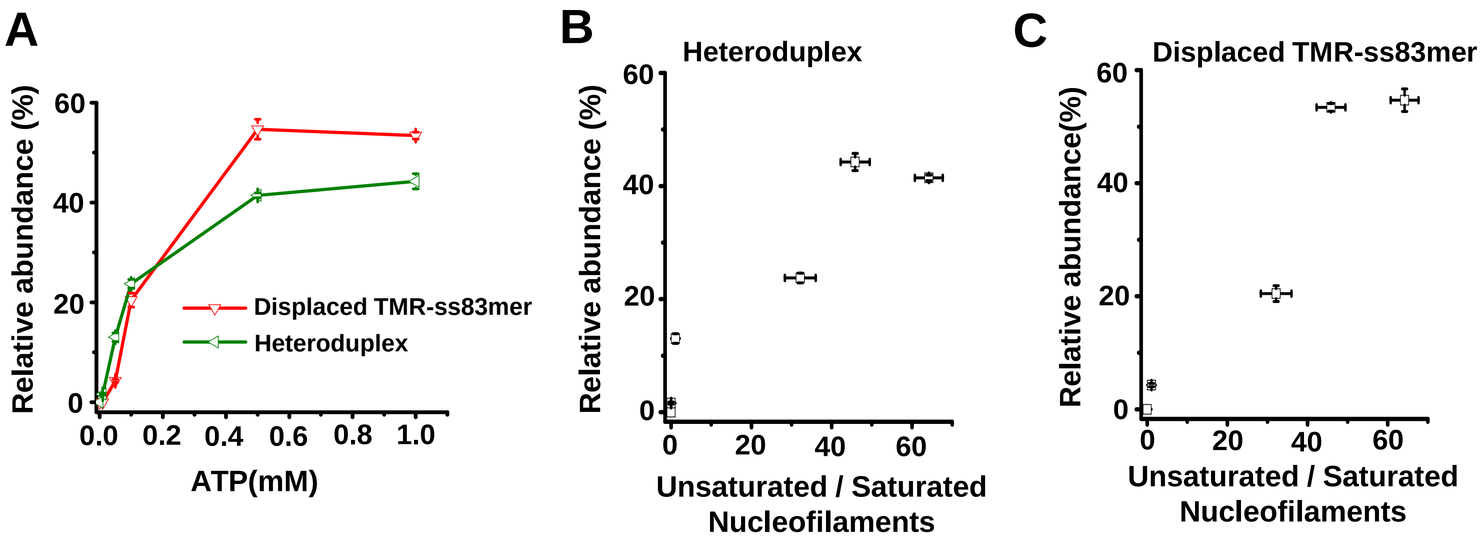

Supplementary Figure S11.The strand exchange efficiency is proportional to the relative abundance of the RecA-unsaturated nucleofilaments.

(**A**) Relative abundance of the products of DNA strand exchange reactions plotted against ATP concentration.

(**B, C**) The correction of the heteroduplex (**B**) and displaced TMR-ss83mer(**C**) with the ratio of the unsaturated nucleofilaments to the saturated nucleofilaments.

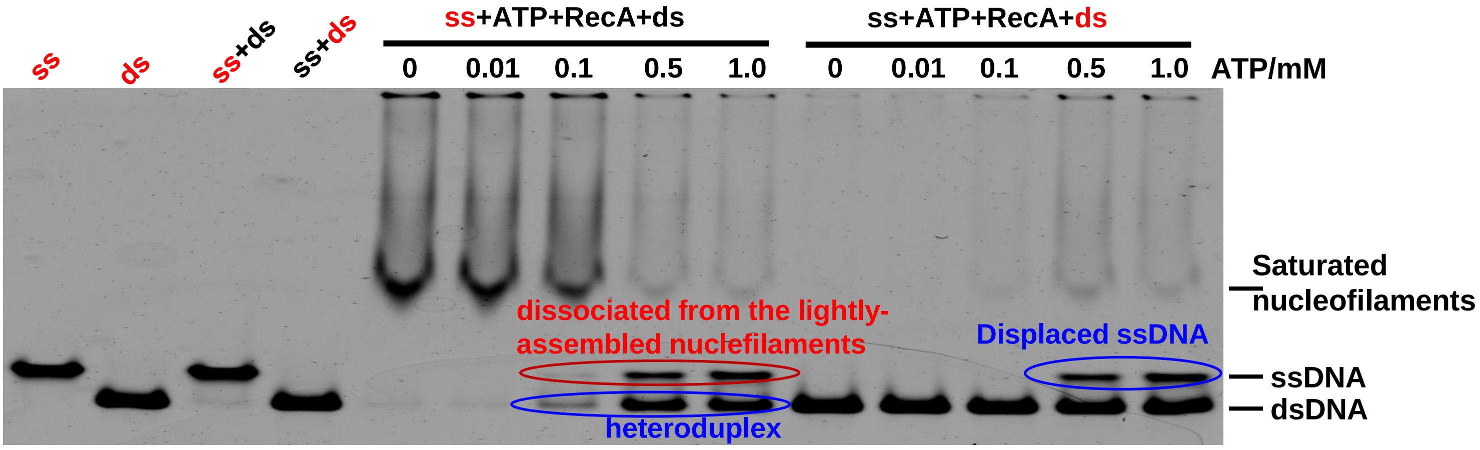

Supplementary Figure S12.The strand exchange mediated by the lightly-assembled nucleofilaments rather than RecA-saturated nucleofilaments is confirmed using PAGE analysis

Electropherograms obtained from native gradient PAGE (4% + 16%) analysis of the mixture of RecA (3.0 μM), ss83mer (20nM), and ds83mer (20 nM) in the presence of varying concentration of ATP (0-1.0 mM), showing ATP-dependent strand exchange. Red letters (see top of panel) indicated the fluorescent labeling of the DNA by Cy5, and black letters (see top of panel) indicated the un-labeling of the DNA. Of note, at 0.5-1.0 mM ATP, the RecA-saturated nucleofilaments significantly reduced and RecA unbound-ssDNA appeared accompanying with the presence of heteroduplex, suggesting the dissociation and instability of the lightly-assembled nucleofilaments during PAGE analysis. Overall, the results further confirmed that the DNA strand exchange was mediated by the lightly-assembled nucleofilaments.

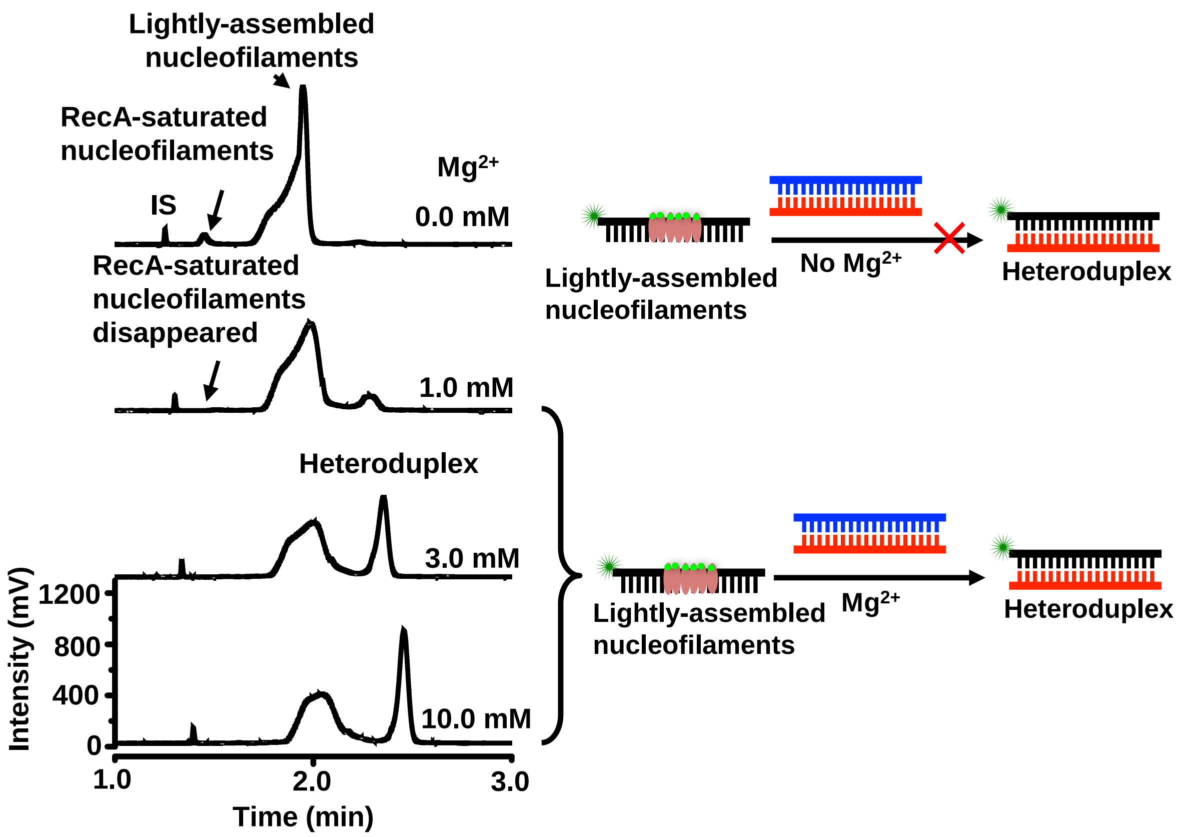

Supplementary Figure S13. **Mg^{2+} diminishes the RecA-saturated nucleofilaments but promotes DNA strand exchange.**

Electropherograms of CE-LIFP analysis of the mixtures of strand exchange reactions in the absence and presence of Mg^{2+}, showing that magnesium ions are indispensable for DNA strand exchange. The reactions contained 3.0 μM RecA, 10 nM TMR-ss83mer,10 nM unlabeled ds83mer, 1.0 mM ATP, and Mg^{2+} at indicated concentrations. Only after undergoing strand exchange, heteroduplex (TMR-ds83mer) could be observed.

Supplementary Figure S14

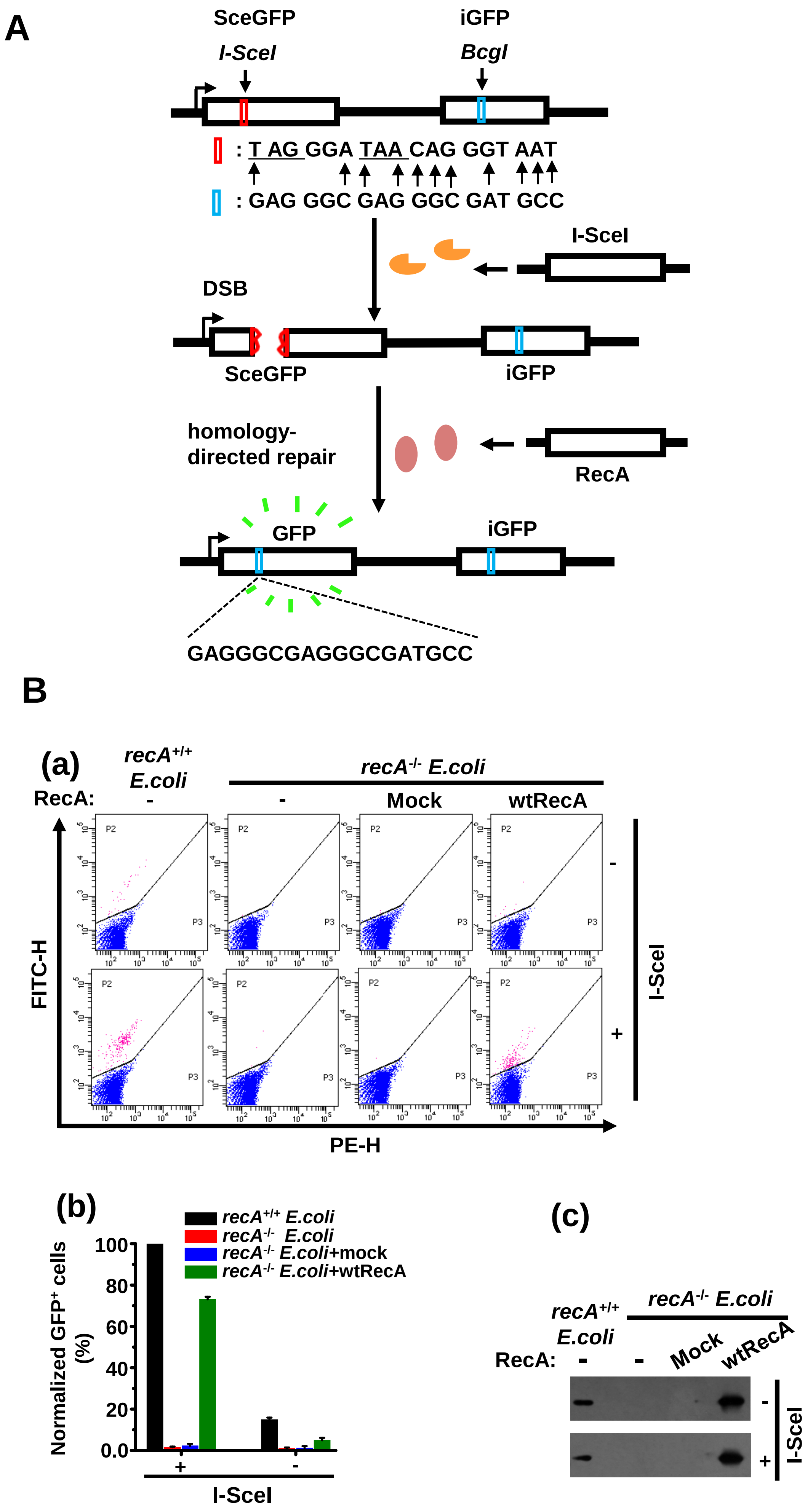

Supplementary Figure S14. *In vivo E. coli* homologous recombination assay.

(**A**) Schematic illustration of the fluorescent reporter (GFP) for homology-directed repair of DSB. SceGFP was obtained from EGFP with the sequence from 97 to 114 (BcgI site) replaced by a rare I-SceI recognition sequence. The full sequence of iGFP was identical to that of the N terminal 500nts of EGFP. The detail see the **Experimental Procedure** section: ***In vivo E. coli* homologous recombination assay**.

(**B**) Fluorescence-activated cell sorter (FACS) analysis of *E. coli* HR efficiency of *recA*$^{+/+}$ and RecA knockout (*recA*$^{-/-}$) *E. coli* strains. (a) FACS counting, (b) the normalized percentage of GFP^{+} cells, and (c) the expression of RecA obtained from western-blotting analysis.

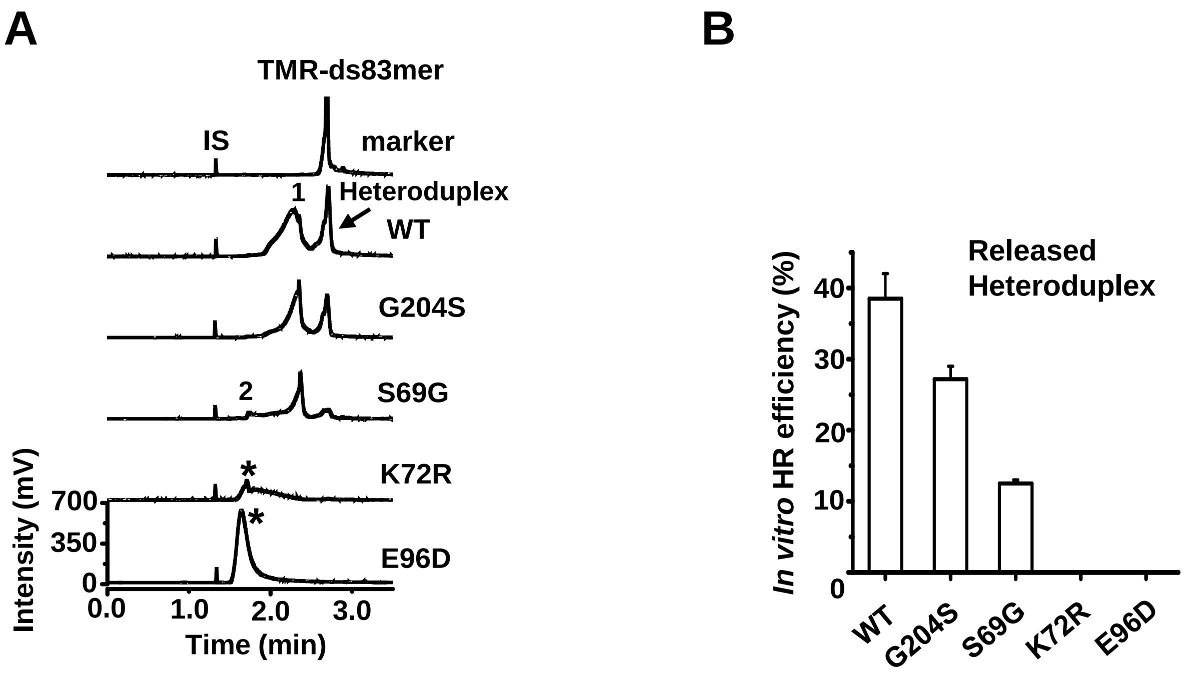

Supplementary Figure S15. *In vitro* strand exchange of four RecA mutants (G204S, S69G, E96D, and K72R).

(**A**) Electrophoregrams obtained from CE-LIFP analysis of the *in vitro* strand exchange products as mediated with the mutated RecA proteins. *Exact peak identity remains unknown, probably due to a mixture of RecA-saturated three-stranded complex and RecA-saturated ssDNA filaments. IS represents the internal migration marker. Peaks 1 and 2 represent the lightly-assembled and moderately-assembled nucleofilaments, respectively.

(**B**) The obtained *in vitro* DNA strand exchange efficiency. The DNA strand exchange reactions involved with 3.0μM RecA,10 nM TMR-ss83mer, 10 nM unlabeled ds83mer, and 1.0 mM ATP. The reactions proceeded for 10 min at 37 °C.

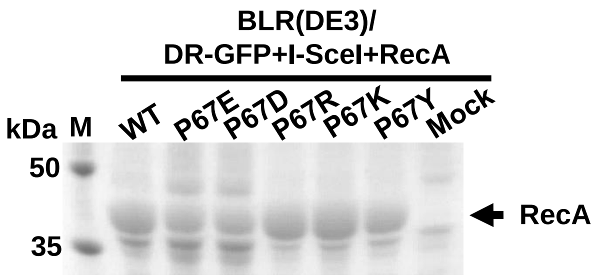

Supplementary Figure S16. The overexpression of P67-mutated RecA proteins.
SDS-PAGE (12 %) analysis of P67-mutated RecA protein expression in transfected *E. coli* strains.

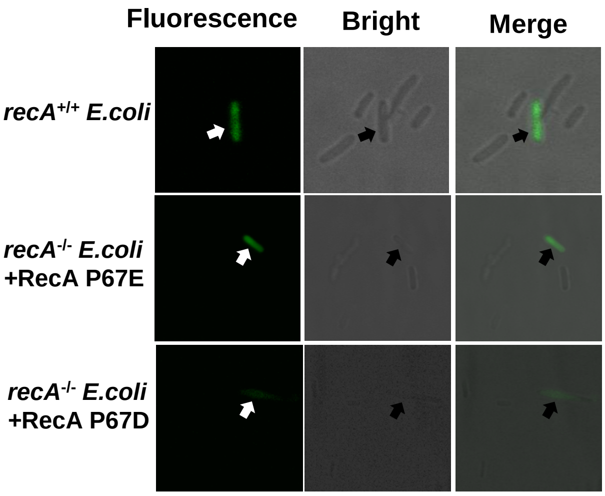

Supplementary Figure S17. RecA mutants (P67D and P67E) that preferentially form the unstaturated nucleofilaments can mediate HR *in vivo* as shown by the fluorescence imaging of reporting *E. coli* strains.

The confocal florescent imaging of RecA$^{-/-}$ *E. coli* strains with overexpression of RecAP67D, or RecAP67E, showing the efficient HR repair *in vivo*.
